# Isolated *Bacillus subtilis* strain 330-2 and its antagonistic genes identified by the removing PCR

**DOI:** 10.1038/s41598-017-01940-9

**Published:** 2017-05-11

**Authors:** Zahoor Ahmad, Jia Wu, Lulu Chen, Wubei Dong

**Affiliations:** Department of Plant Pathology, College of Plant Science and Technology and the Key Lab of Crop Disease Monitoring & Safety Control in Hubei Province, Huazhong Agricultural University, Wuhan, Hubei Province China

## Abstract

Plant growth-promoting bacteria (PGPB) may trigger tolerance against biotic/abiotic stresses and growth enhancement in plants. In this study, an endophytic bacterial strain from rapeseed was isolated to assess its role in enhancing plant growth and tolerance to abiotic stresses, as well as banded leaf and sheath blight disease in maize. Based on 16S rDNA and BIOLOG test analysis, the 330-2 strain was identified as *Bacillus subtilis*. The strain produced indole-3-acetic acid, siderophores, lytic enzymes and solubilized different sources of organic/inorganic phosphates and zinc. Furthermore, the strain strongly suppressed the *in vitro* growth of *Rhizoctonia solani* AG1-IA, *Botrytis cinerea*, *Fusarium oxysporum*, *Alternaria alternata*, *Cochliobolus heterostrophus*, and *Nigrospora oryzae*. The strain also significantly increased the seedling growth (ranging 14–37%) of rice and maize. Removing PCR analysis indicated that 114 genes were differentially expressed, among which 10%, 32% and 10% were involved in antibiotic production (e.g., *srfAA*, *bae*, *fen*, *mln*, and *dfnI*), metabolism (e.g., *gltA*, *pabA*, and *ggt*) and transportation of nutrients (e.g., *fhu*, *glpT*, and *gltT*), respectively. In summary, these results clearly indicate the effectiveness and mechanisms of *B. subtilis* strain 330-2 in enhancing plant growth, as well as tolerance to biotic/abiotic stresses, which suggests that the strain has great potential for commercialization as a vital biological control agent.

## Introduction

Plant growth-promoting bacteria (PGPB)^1^ colonize the plant rhizosphere, which enhances the plant growth and control of soil-borne diseases^2^. The PGPB associated with plant roots are often classified by the locations where they colonize, i.e., rhizosphere and endosphere^3^. The PGPB living in the rhizosphere and endosphere are known as rhizobacteria and endophytes, respectively. *Bacillus* species are important members of PGPB, which induce plants to tolerate abiotic and/or biotic stresses in a comprehensive manner^4^. *Bacillus* spp. have also been commercialized as biofertilizers and biocontrol agents^5^. The endophytic *Bacillus* spp. do not cause any visible damage or morphological alterations to the host. Therefore, these bacteria can be beneficial for the survival of the host species against environmental stresses and microbial competition^6, 7^. Furthermore, these bacteria may promote the growth of the plant through the formation of nodules with non-specific hosts^8^.

*Bacillus* spp. produce indole-3-acetic acid (IAA), which helps with nitrogen fixation from the atmosphere, siderophore production, solubilization of potassium (K), zinc (Zn), and phosphate (P) from the soil, and increasing the soil porosity^7, 9^ Along with the micro- and macro-nutrient supply, endophytic *Bacillus* spp. protect the plants from pathogens and play roles as antagonists^7^. They inhibit the activities of pathogens by producing diverse antimicrobial compounds, including siderophores^10^, hydrolytic enzymes^11^ and antibiotics^12^, volatile organic compounds (VOCs)^13^, and lipopeptides^14^ that are associated with the observed biocontrol activity against plant pathogens^15, 16^.

*Rhizoctonia solani* Kühn (teleomorph: *Thanatephorus cucumeris* (Frank) Donk) is one of the most prevalent soil-borne pathogen, which causes a significant economic losses in several economically important crops such as maize, rice and soybean^17^. Banded leaf and sheath blight (BLSB), caused by *R. solani*, is the most threatening disease, which may cause complete crop failure^18^. *B. cinerea* is an aggressive pathogen with a wide host range and uses multiple weapons to invade its host plants^19^, while *F. oxysporum* causes two major diseases of maize such as Fusarium ear rot and Gibberella ear rot, both of which can result in mycotoxic contamination of maize grains^20^. Under temperate and moist conditions, *C. heterostropus* causes southern leaf blight on corn and have devastating effect on other major cereal crops^21^. In addition to these devastating diseases, there are many other economically important diseases such as, rice grain spot and root rot of maize caused by *N. oryzae* under stress conditions^22^. On the other hand, *A. alternata* is one of the most common contaminating fungal pathogen, detected in cereal grains before harvest and may contribute to decrease in grain quality^23^.

The isolation and identification of differentially expressed genes was performed using several methods, including differential analysis of library expression (DAZLE)^24^, representational difference analysis (RDA)^25^, differential display and related techniques^26^, enzymatic degradation subtraction^27^, techniques involving physical removal of common sequences^28^, linker capture subtraction^29^, and suppression subtractive hybridization (SSH)^30^. These methods are vital but also possess some intrinsic drawbacks, such as the fact that the sequence must be known in advance. This leads to the generation of many false positives, sequencing that produces very short sequences, lack of reproducibility, and post-sequencing data analysis expenses^31–33^. Recently, a novel technique called removing polymerase chain reaction (R-PCR) has been used to efficiently remove the undesirable genes from a gene population^33^. The R-PCR reaction is the reverse process of a PCR. In PCR, the desired genes are amplified cycle by cycle, whereas in R-PCR, the undesired genes get removed cycle by cycle. The R-PCR permits rapid identification of differentially expressed genes, while excluding the false positive and false negative clones. Therefore, we performed this experiment to identify the antagonism-related genes from *B. subtilis* strain 330-2 using the most advanced R-PCR technique.

Therefore, one endophytic *Bacillus* was isolated from a rapeseed that had broad-spectrum antagonistic activity against several phytopathogenic fungi, including *R. solani*, *F. oxysporum*, *B. cinerea*, *A. alternata*, *C. heterostrophus* and *N. oryzae*. This study was aimed at (1); Isolation, identification, and characterization of the bacterial *B. subtilis* strain 330-2. (2); Evaluation of the antagonism of *B. subtilis* 330-2 on different plant pathogens. (3); Identification of the antagonism-related genes using R-PCR.

## Results

### Identification and properties of the strain

**Figure 1:**
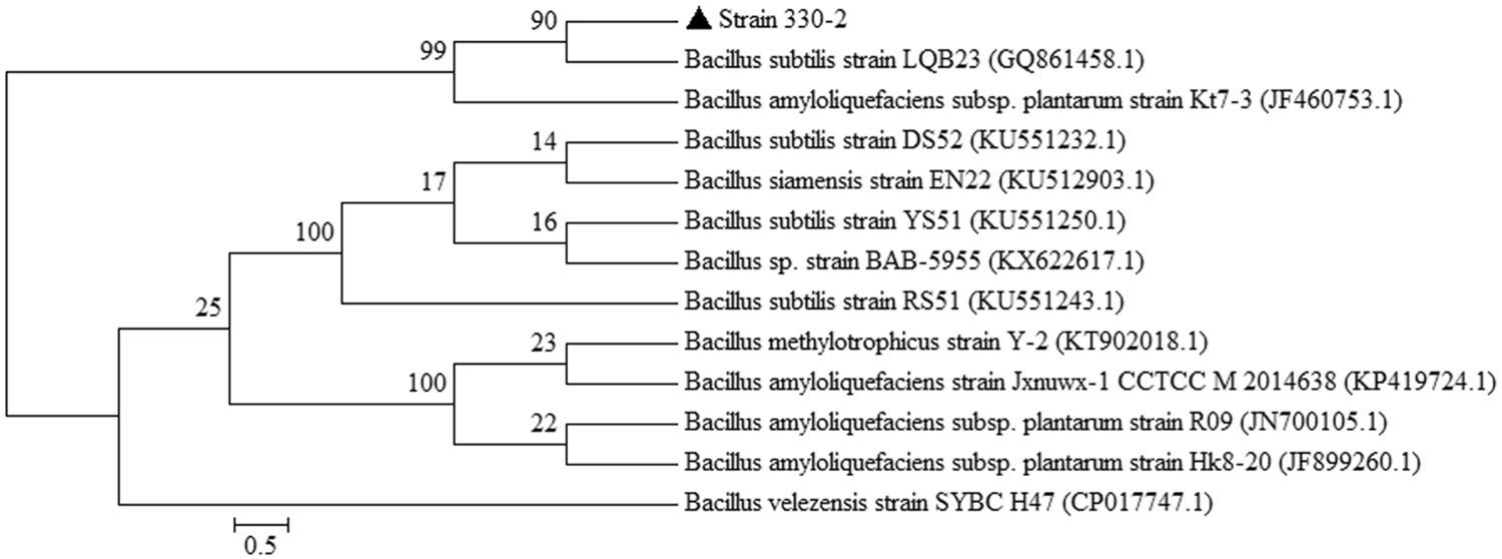
An amplified 16S rDNA gene fragment from the isolated strain 330-2 was sequenced and blast searched through NCBI database. Closely related sequences were downloaded and aligned. These sequences were analyzed using the Maximum likelihood method. The GenBank accession number of each isolate is given in parentheses. Bootstrap values based on 1000 replicates are shown next to the branches.

The isolated strain was catalase-positive, gram-positive, rod-shaped, aerobic, and motile and had distinct fermentation profiles for different carbon sources (Supplementary Table 1). Based on the observed phenotypic characteristics, the isolated strain was grouped into the genus *Bacillus*. Furthermore, it exhibited high levels of similarity (99%) to the closest known species in the database. Phylogenetic analysis of the 16S rDNA gene sequence revealed that the isolated 330-2 strain was *Bacillus subtilis*. In addition to *B*. *subtilis*, another species of the genus *Bacillus, B. amyloliquefaciens*, showed (99%) similarity with the strain during the BLAST search. The phylogenetic tree analysis using Mega5 version 5.2 showed that strain 330-2 was more closely related to *B. subtilis* and *B. amyloliquefaciens* (Fig. 1). To further confirm the strain, a BIOLOG test was performed.

### BIOLOG identification of the bacterial strain

**Figure 2:**
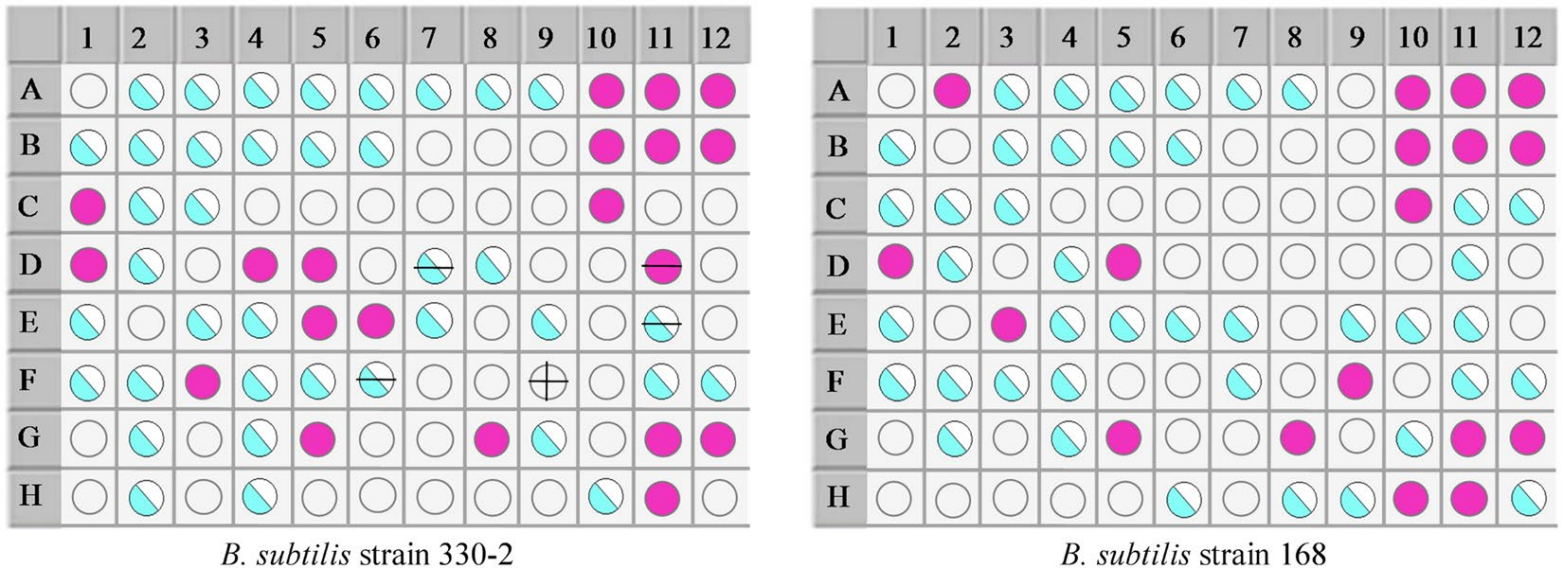
The BIOLOG detection results showing the comparison of the *B. subtilis* strain 330-2 and *B. subtilis* strain 168. The negative control (A1); the positive control (A10). The well with a faint purple color shows the positive reaction, the half-filled light blue circle shows the moderate reaction, while the blank circle shows the negative reaction.

Phenotypic analyses was done using 96-well BIOLOG GENIII MicroPlates assay. The list of carbon substrates used by *B. subtilis* sp. is presented (Fig. 2 and Supplementary Table 1). The results of the 16S rDNA sequencing and BIOLOG identification indicated that the strain used in this study was *B. subtilis*.

### Production of hydrolytic enzymes and metabolites by bacterial antagonists

Progression of the pink color in the presence or absence of tryptophan in the culture broth showed the production of IAA. Maximum IAA production was recorded as 24.13 μg ml^−1^ in the *B. subtilis* strain 330-2, followed by 2.10 μg ml^−1^ in *Bacillus subtilis* strain 168 broth culture (*B. subtilis* strain 168 was taken as control). The *B. subtilis* strain 330-2 also produced the siderophore through the development of orange halo zones around their spot of inoculation on Chrome-azurol S (CAS) medium, while *B. subtilis* strain 168 did not produce a siderophore.

The *B. subtilis* strain 330-2 showed a positive result for phosphate solubilization by spot inoculation in the solid state of Pikovskaya’s media, but did not solubilize the Di-calcium phosphate (DCP) by spot inoculation on Pikovskaya’s medium. On the plates, *B. subtilis* strain 330-2 P-solubilizing zones around the bacterial colony were 4.2 and 6.3 mm using Tri-calcium phosphate (TCP) and zinc phosphate (ZP), respectively. However, *B. subtilis* strain 168 could not solubilize the phosphate in Pikovskaya’s solid medium. Both strains were tested for phytase production, whereas *B. subtilis* strain 330-2 solubilized calcium and sodium phytate by formation of halo zones around its spot of inoculation (12.7 and 9.6 mm for calcium and sodium phytate, respectively), suggesting the release of free P. The *B. subtilis* strain 168 did not solubilize the sodium phytate, except on calcium phytate-amended medium through the formation of a 6.6 mm halo zone around the bacterial colony.

**Figure 3:**
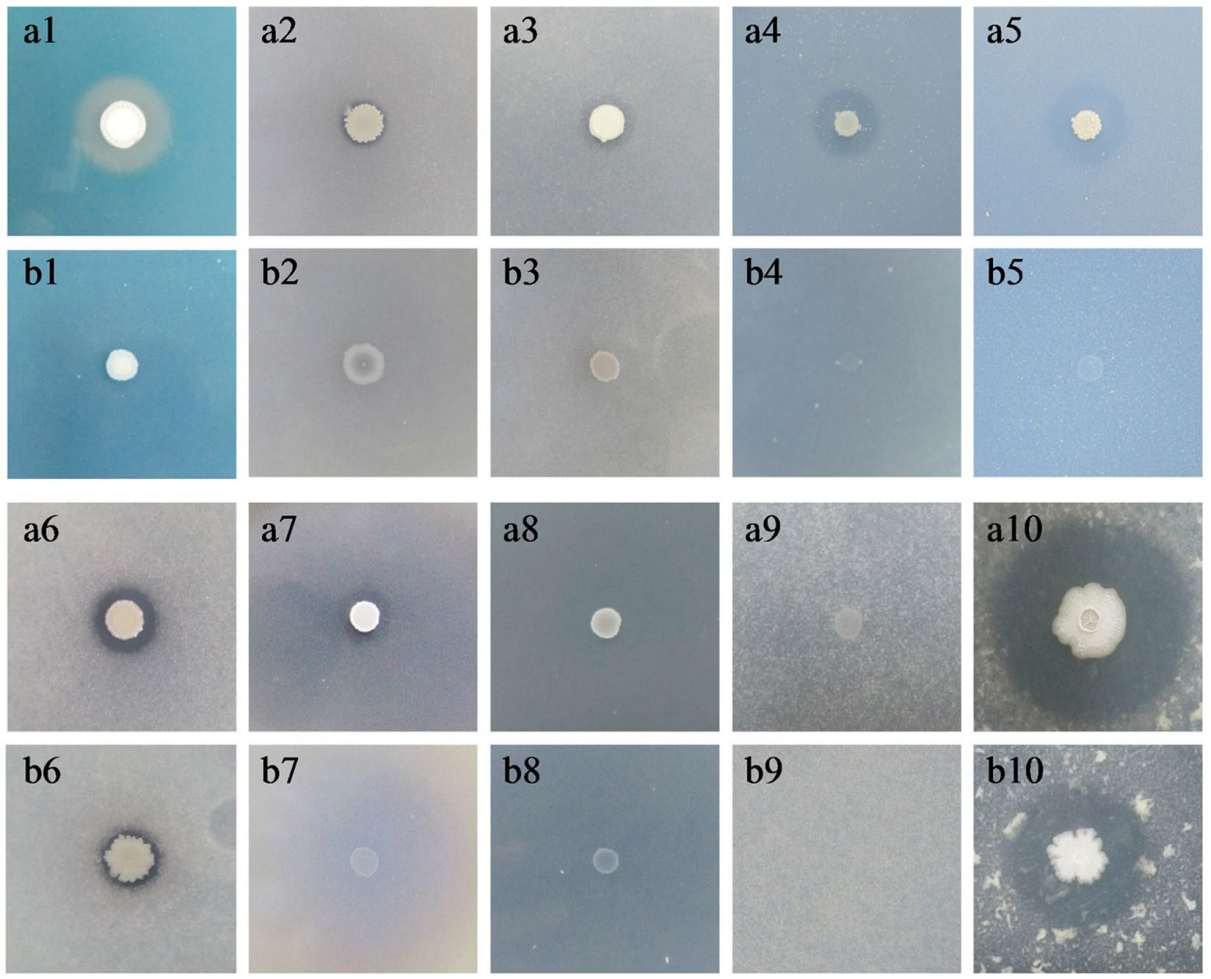
Production of hydrolytic enzymes and metabolites by *B. subtilis* strain 330-2 and *B. subtilis* strain 168. *B. subtilis* strain 330-2 (**a**); *B. subtilis* strain 168 (**b**); Siderophore production (1); Tri-calcium phosphate (2); Zinc phosphate (3); Zinc oxide (4); Zinc phosphate (5); Ca-phytate (6); Na-phytate (7); Laminarinase (8); Cellulase (9) and Protease production (10). All experiments were conducted three times through three independent trials.

The *B. subtilis* strain 330-2 solubilized zinc oxide and zinc phosphate, which led to the formation of clear halo zones of 12.26 and 13.5 mm around the bacterial spot on the respective medium compared to *B. subtilis* strain 168, which did not solubilize zinc oxide and zinc phosphate. Both strains showed positive protease activity by producing clear halo zones of 29.5 mm and 17.8 mm around the spot inoculation. Both strains grew on laminrain azure and cellulose-amended minimal media, except strain *B. subtilis* strain 168, which did not grow on cellulose-amended medium (Table 1 and Fig. 3).

**Table 1:** Plant growth promoting attributes and production of hydrolytic enzymes by *B. subtilis* strain 330-2 and *B. subtilis* strain 168.

Production of	*Bacillus subtilis* strains	
	330-2 (Mean ± SD)	168 (Mean ± SD)
indole-3-acetic acid	(24.13 ± 0.31)^a^	(2.10 ± 0.20)^b^
Siderophore	(17.5 ± 0.80)	(0)
**Phosphate solubilization (Inorganic phosphate)**		
Tri-calcium phosphate	(4.2 ± 0.3)	(0)
Di-calcium phosphate	(0)	(0)
Zinc phosphate	(6.3 ± 0.91)	(0)
**Phosphate solubilization (Organic phosphate/phytase production)**		
Ca-Phytate	(12.7 ± 0.2)^a^	(6.6 ± 0.15)^b^
Na-Phytate	(9.6 ± 0.3)	(0)
**Zinc solubilization**		
Zinc oxide	(12.26 ± 0.49)	(0)
Zinc Phosphate	(13.5 ± 0.26)	(0)
**Hydrolytic activity**		
Protease	(29.5 ± 0.55)^a^	(17.8 ± 0.3)^b^
β-1, 3-glucanase	+	+
β-1, 4-glucanase	+	−

Values are the mean of three replicates with standard deviation. All experiments were conducted in triplicates through three independent trials. Data were statistically analyzed by analysis of variance (ANOVA) and least significant difference (LSD) test at (*P* < 0.05%) level of significance −negative; +positive.

### *In vitro* antagonistic activity assays

**Figure 4:**
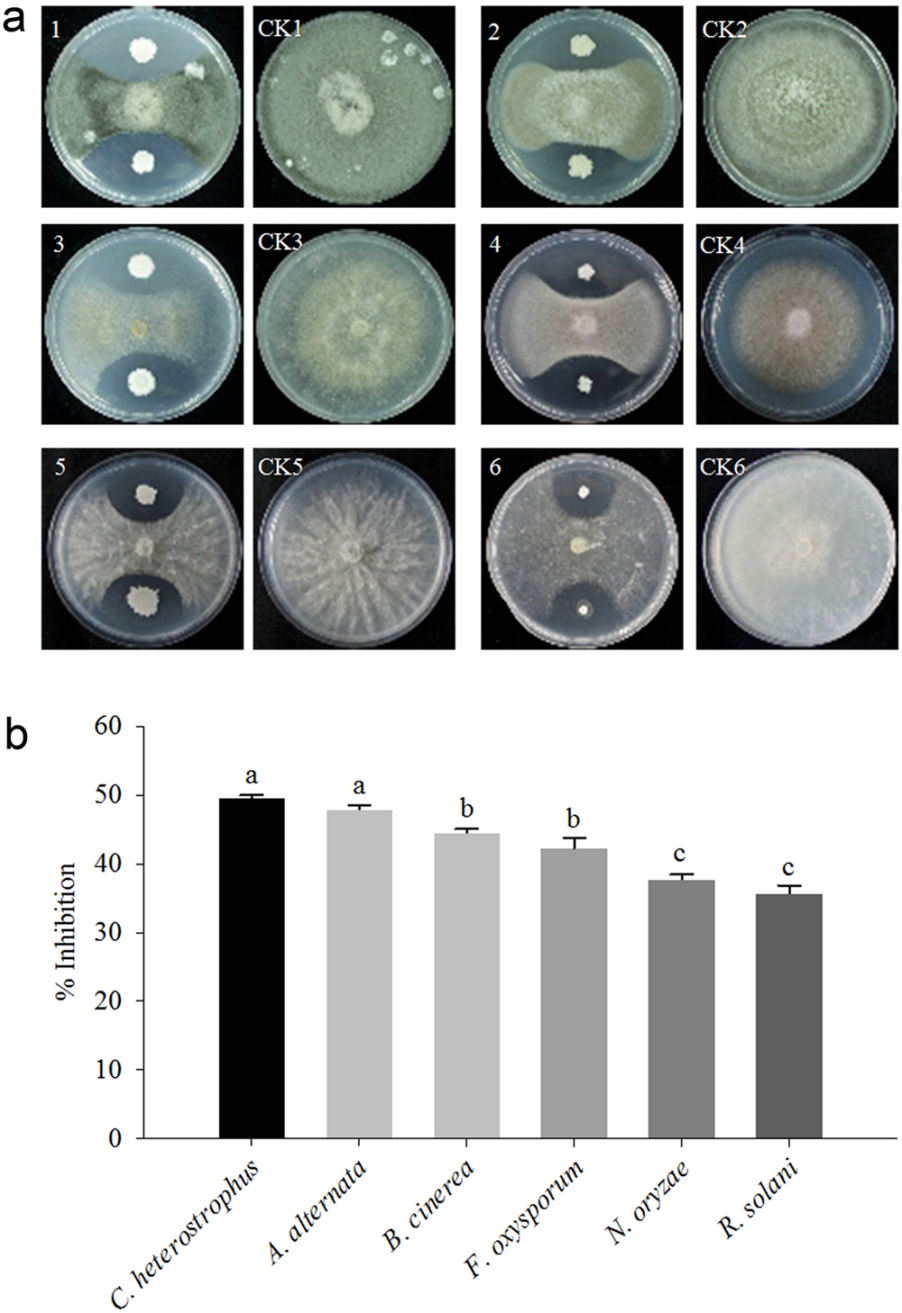
Effects of *B. subtilis* strain 330-2. (**a**) Antagonistic effects of *B. subtilis* strain 330-2 against fungal pathogens; *C. heterostrophus* (1); *A. alternate* (2); *B. cinerea* (3); *F. oxysporum* (4); *N. oryzae* (5); *R. solani*AG1 (6); and with their respective control (CK) on dual culture. (**b**) % inhibition of *B. subtilis* strain 330-2 against fungal pathogens. Data were statistically analyzed and the small alphabetical letters (**a, b, c**…) above the mean bars show the significant differences (*P* < 0.05) among treatments against different fungal pathogens. Each assay was performed in triplicate.

The *B. subtilis* strain 330-2 illustrated the highest antifungal activity against all the phytopathogens listed (Fig. 4). The data on mycelium growth was recorded based on the respective control treatment (4–7 days) and revealed considerable reductions in the growth of fungal mycelia under the influence of *B. subtilis* strain 330-2. The maximum inhibition (%) in the radial growth by *B. subtilis* strain 330-2 was 49.53% for *C. heterostrophus*, followed by 47.96% for *A. alternata*, 44.57% for *B. cinerea*, 42.25% for *F. oxysporum*, 37.74% for *N. oryzae*, and 36.28% for *R. solani*. When compared with the controls, each of the treatments showed significant difference (*P* < 0.05).

### Plant growth enhanced by *B. subtilis* strain 330-2

**Figure 5:**
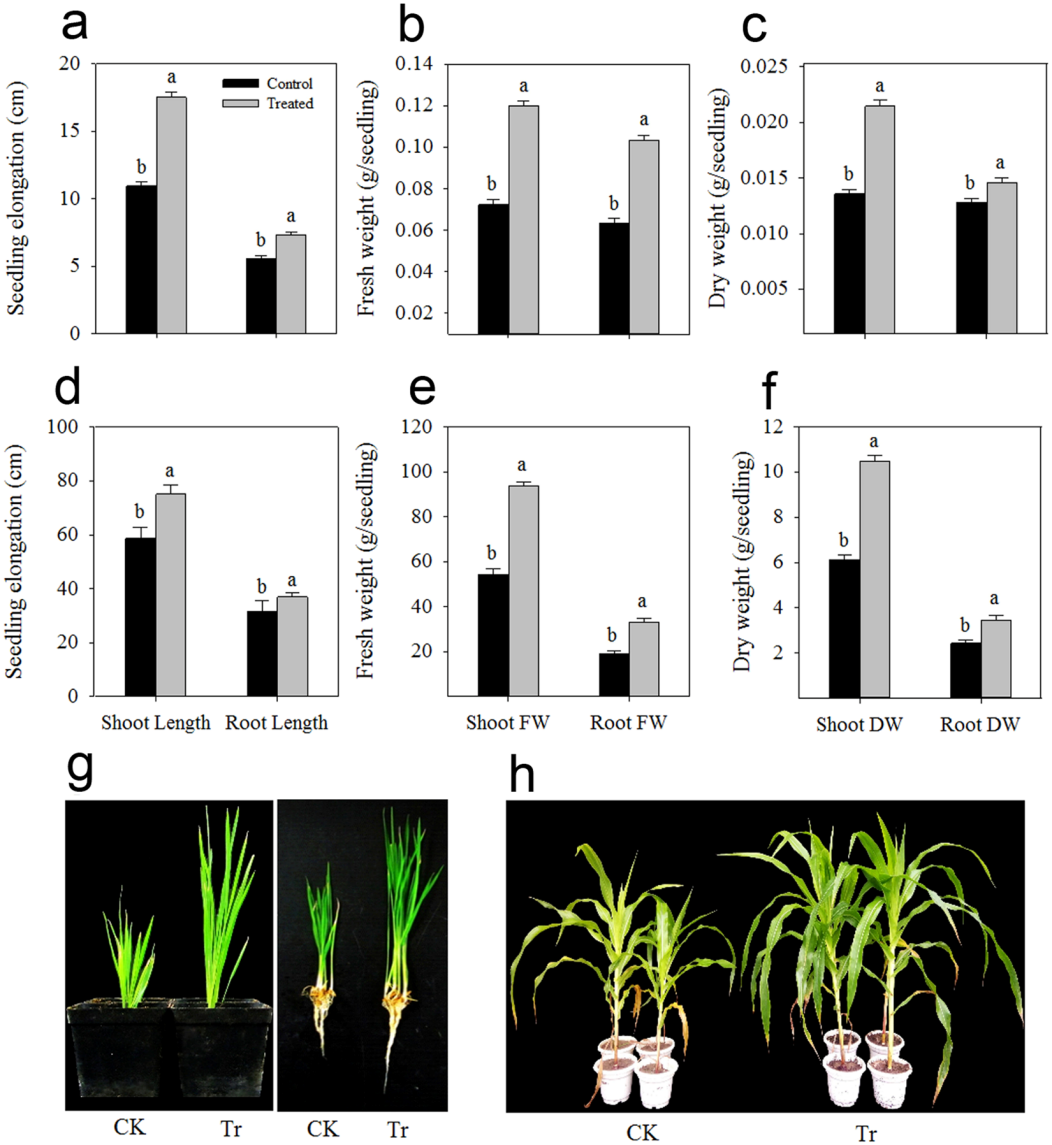
Effect of *B. subtilis* strain 330-2 on the seedling growth performance of rice and maize. (**a** and **d**) Seedling elongation of rice and maize, (**b** and **e**) seedling fresh weight of rice and maize, (**c** and **f**) seedling dry weight of rice and maize, and (**g** and **h**) pictorial view of 3-weeks and 40 days old rice and maize seedling in the control and *B. subtilis* strain 330-2 treatment, respectively. Data were statistically analyzed and the vertical bars above indicate the standard error of three replicates. Small alphabetical letters (**a, b**…) above the mean bars show the significant differences (*P* < 0.05) among the treatments within specific parameters. FW: Fresh weight, DW: Dry weight, CK: Control, Tr: Treated with *B. subtilis* strain 330-2.

The *B. subtilis* strain 330-2 was chosen to determine its beneficial effects on rice and maize growth under greenhouse conditions. The performance of the rice and maize shoots were far better than the controls when bio-primed with the *B. subtilis* strain 330-2 (Fig. 5a–c and g). *B. subtilis* strain 330-2 was found to enhance the shoot and root length, shoot and root fresh weight and shoot and root dry weight of rice significantly (*P* < 0.05) by 37.41, 23.90, 39.81, 38.31, 36.79 and 12.21%, respectively, compared with the control. The respective traits for maize were 21.98, 14.19, 41.95, 42.09, 41.66 and 29.15% under the influence *B. subtilis* strain 330-2 (Fig. 5d–f and h).

### Biosafety and plant growth promoting effect of *B. subtilis* strain 330-2

The addition of *B. subtilis* strain 330-2 resulted in significantly (*P* < 0.05) increased plant height, as well as an increase in the fresh and dry weight (Supplementary Fig. 1a–c and d) compared to the non-inoculated plants. The massive inoculation of the *B. subtilis* strain 330-2 did not have a detrimental effect.

### Generation of differentially expressed clones by R-PCR

**Figure 6:**
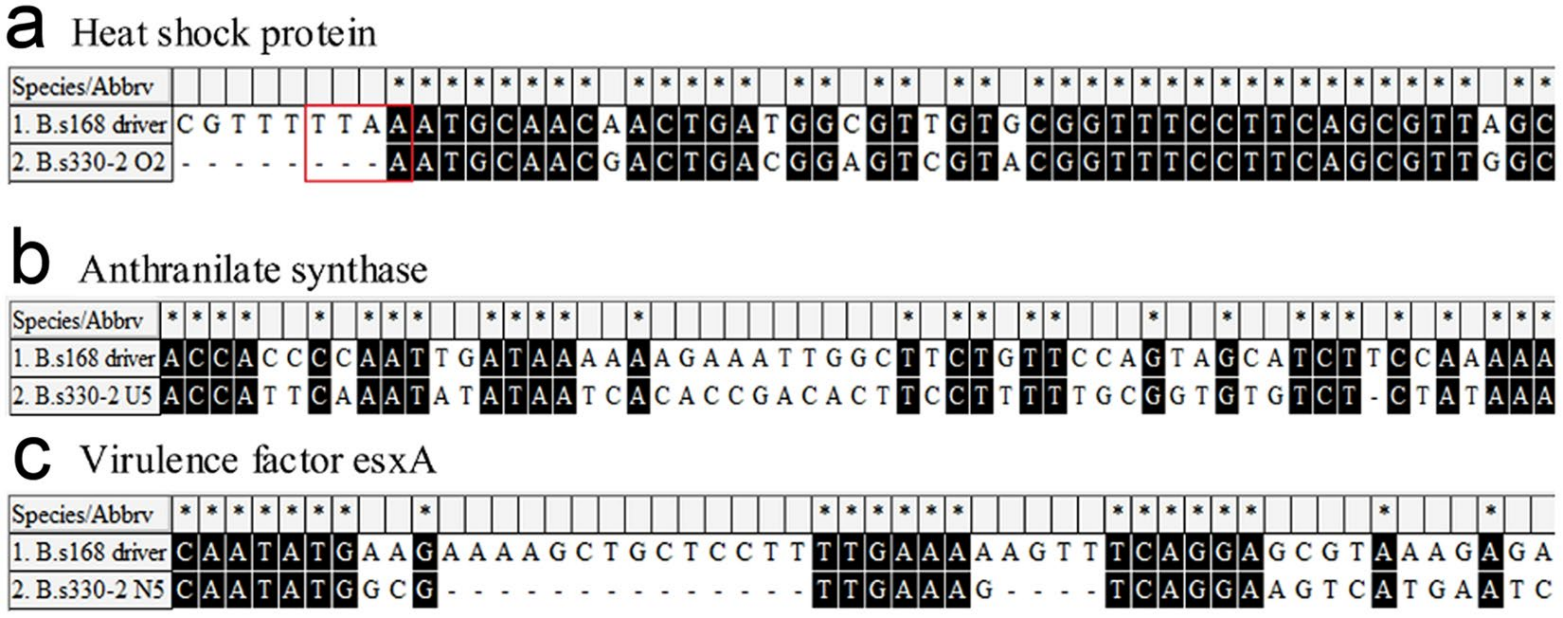
The driver in R-PCR could not eliminate the tester in three cases: (**a**) the recognition cutting sites of the *Mse*I and *ApeK*I (in red box) have mutations, (**b**) the tester contains enough bases that do not match the driver and (**c**) the sequence of the tester or driver is partially absent (or completely absent in the driver). *Identical.

The sequence identities between the tester and driver were found to be <87% as determined under diversifying differentiation with a specified selection^33^. Therefore, 87% identity was used as one of the evaluating criteria to determine R-PCR cloned genes. In the recent study, the R-PCR efficiency was estimated to be 92.10% (Supplementary Table 3). To underpin the removal mechanisms of R-PCR at the genomic DNA sequence level, both the tester and driver sequences were compared using MEGA 5 version 5.2 and it was found that, in the three main cases, the driver could not eliminate the tester (Fig. 6 and Supplementary Fig. 3). The sequence identities of the R-PCR cloned genes between tester and driver are presented (Supplementary Table 4).

### Identification and functional categorization of differentially expressed genes in *B. subtilis* strain 330-2

**Figure 7:**
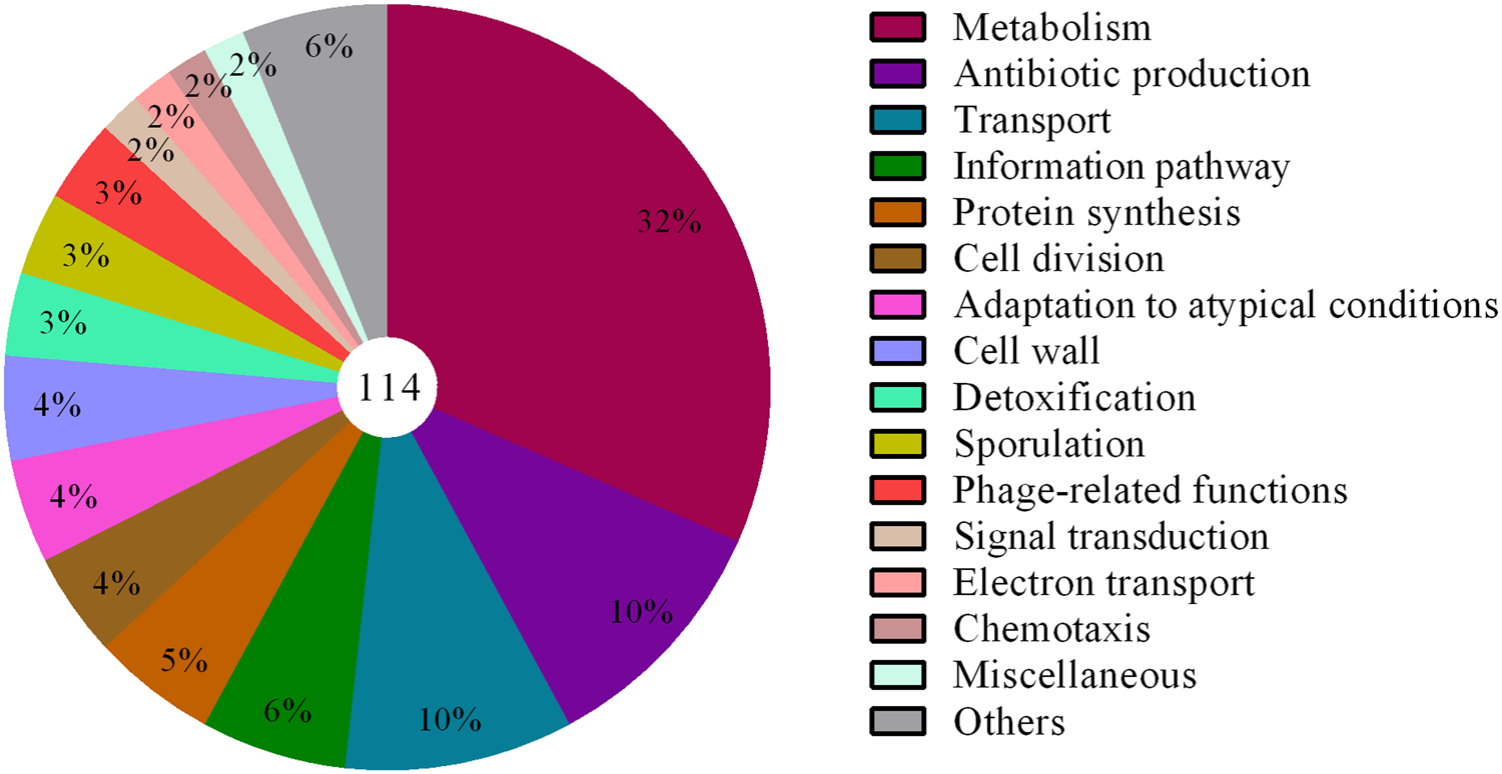
Distribution in various functional categories of differentially expressed genes by R-PCR. The percentage of each group is indicated in each category, and the total numbers of genes are presented in the center of the graph.

Gene Ontology (GO) was performed to explore the functions of the differentially expressed genes. In total, 114 differentially expressed genes were assigned to three main functional categories (biological process, cellular component and molecular function) (Supplementary Table 2 and Supplementary Fig. 2a–c). We focused on the groups that were associated with stress tolerance, defense and plant growth enhancement (Fig. 7).

In the present study, five genes that were associated with cell motility (*hag*, *mcpA*), biofilm formation and root colonization (*srfAA*, *wapA*, *spo0A*) were differentially expressed under treatment with *B. subtilis* strain 330-2. *B. subtilis* strain 330-2, which was observed to regulate the expression of different NRPSs, including surfactin (*srfAA*), bacillaene (*bae*) and fengycin (*fen*). In addition to NRPS, *B. subtilis* strain 330-2 triggered the production of secondary metabolites, including antibiotics such as macrolactin (*mln*), difficidin (*dfn*), iturin A (*ituA*), bacillibactin (*dhbF*), penicillin binding protein 2B (*pbpB*) and beta-lactamase (*penP*), which are important manifestations of a plant’s defense mechanism and can cope with competing microorganisms and inhibit the growth of phytopathogenic fungi or bacteria.

**Figure 8:**
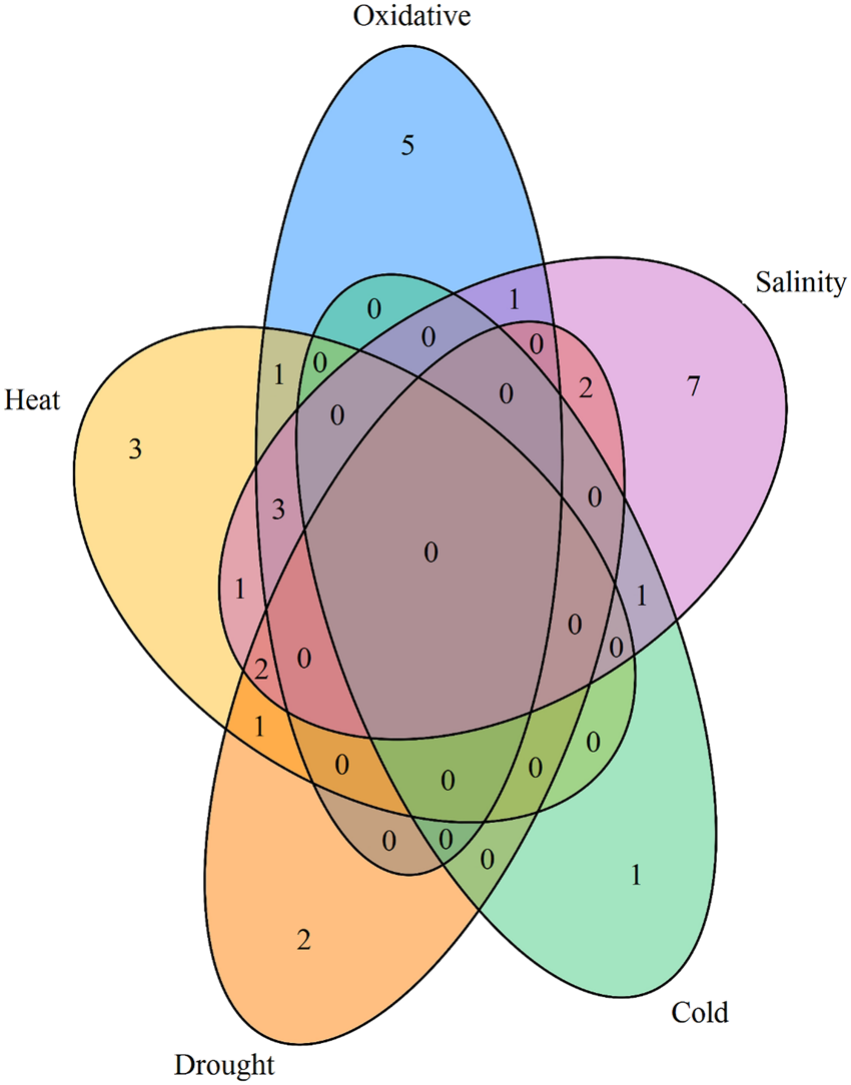
Venn diagram indicating the common genes shared by salinity, oxidative, heat, cold and drought stresses. In total, 12 common differentially expressed genes were identified in all the five abiotic stresses.

Different genes associated with tolerance to heat (elongation factor Tu; aspartokinase II; and dihydroorotase, *pyrC*), salinity (cardiolipin synthase, *ywiE*; glutaminase-1, *ybgJ*; phosphoglyceratemutase, *gpmI*; 4-hydroxy-3-methylbut-2-enyl diphosphate reductase, *ispH* and glutamate synthase (NADPH/NADH), *gltA*), drought (cystathionine beta-lyase and cysteine desulfhydrase) and cold stresses (sporulation cortex protein, *coxA*) were differentially expressed in *B. subtilis* strain 330-2. Interestingly, NADH-dependent butanol dehydrogenase A (*yugJ*) and formate dehydrogenase (*yjgC*) are commonly involved in tolerance to heat, salinity and oxidative stresses. Likewise, glutamate symporter (*gltT*) and S-adenosylmethionine synthase (*metK*) are commonly involved in salinity and drought stress (Fig. 8). These results clearly suggest the beneficial role of *B. subtilis* strain 330-2 in enhancing the plant’s tolerance to different abiotic stresses.

Five genes involved in abiotic stress tolerance, e.g., *pabA*, the precursor of tryptophan (indole-3-acetic acid synthesis); bioA, responsible for biotin biosynthesis; *narI*, nitrate reductase production; *pckA* was shown to be involved in nitrogen fixation, *ispH* is responsible for isoprenoid biosynthesis, were found to be differentially expressed in *B. subtilis* strain 330-2 were found to be differentially expressed in *B. subtilis* strain 330-2. All these processes are critical for the normal growth and development of plants.

Four genes involved in cellulose degradation (*epsB*, *ywqC*, *ydjE* etc.), as well as xylan transport and utilization (*xynA*), were differentially expressed in the *B. subtilis* strain 330-2 (Supplementary Table 2).

## Discussion

The present study was conducted to isolate an antagonistic *B. subtilis* strain 330-2 that can efficiently control a wide range of phytopathogens. The biochemical, physiological and phylogenetic analyses of the 16S rDNA gene sequences and BIOLOG test confirmed that strain 330-2 was *B. subtilis*. The BIOLOG micro plate assay has mainly been used for bacterial biocontrol agents in unraveling their specific carbon sources. The isolation of endophytic *Bacillus* species from various crops has been extensively studied^7, 34^. *B. subtilis* strain 330-2 produced significant amounts of IAA (24.13 µg ml^−1^), which might play an important role in seedling growth and development^35^. Eighty percent of the soil rhizosphere bacteria can produce IAA, whereas almost all (98%) of the PGPR strains isolated from the plant rhizosphere were able to produce IAA^36^. Significant amounts of *in vitro* IAA production by *Bacillus* spp. have also been documented by Singh *et al*.^37^. The production of siderophores by *B. subtilis* strain 330-2 demonstrated its antagonistic nature against various pathogens along with plant growth regulation. In the present study, *B. subtilis* strain 330-2 was found to produce phytase and was a good solubilizer of phosphate and zinc^38^. Phytase has been identified and characterized previously from several gram-positive and gram-negative soil bacteria, such as *B. subtilis* ^39^, *B. laevolacticus*, *Pseudomonas* sp.^40, 41^, and *Enterobacter* sp.^42^. Extracellular phytase from *B. amyloliquefaciens* FZB45 enhanced the growth of maize seedlings under *in vitro* conditions and, in another study, phytase producing bacterial isolates promoted the plant growth of Indian mustard^43, 44^.

Because of the restricted radial growth of the selected phytopathogens, formation of inhibition zones by *B. subtilis* strain 330-2 might be linked to the production of antimicrobial compounds such as antibiotics, cellulase, laminarinase, protease, siderophore production, as well as nutrient competition^37^. Notably, *B. subtilis* strain 330-2 secreted a complex of hydrolytic enzymes, including β-1,3-glucanase, β-1,4-glucanase and proteases, which possibly degrade the contents of the fungal cell wall, such as β-1,3-glucan and glucosidic bonds. However, rare literature is available on the production of hydrolytic enzymes by microorganisms^45, 46^. Therefore, it is assumed that the cell wall lysis of the pathogenic fungi is due to the coordinated action of hydrolytic enzymes, such as laminarase, cellulase and protease^47^. In the present study, *B. subtilis* strain 330-2 was quite effective against all six of the pathogens tested, indicating its potential efficacy as a biocontrol agent and suggesting its use as a bioinoculant for banded leaf sheath in both rice and maize.

The efficiency of the isolated strain in enhancing plant growth was further assessed in a screen-house experiment. *B. subtilis* strain 330-2 was found to produce a considerable amount of IAA and significantly enhance the growth of plants compared with the control. For instance, compared with the control, the *B. subtilis* strain 330-2-treated rice/maize seedlings showed 12–34% enhancements in seedling elongation and biomass accumulation (Fig. 5a–f). It has been stated that the endophytic *Bacillus* (*B. megaterium* strain HNSQJYH124*, B. subtilis* strain HNSQJYH135, and *B. atrophaeus* strain HNSQJYH170) significantly enhanced root length, shoot/root fresh and dry weight and chlorophyll content in wheat seedlings when inoculated with *Bacillus* ^7^. *B. subtilis* and *P. aeruginosa* increased the plant growth of African spinach, okra and tomato in terms of shoot length and dry biomass^48^. More recently, it was also found that the inoculation of plants with PGPB enhanced the root colonization and plant growth^4, 49^. The mechanisms underlying the promotion of plant growth are diverse, including synthesis of phytohormones and signaling molecules, improvement of the plant mineral nutrition and production of siderophores and volatile compounds^50^. It has been reported that *Bacillus* spp. can elicit induced systemic resistance (ISR), which may result in the promotion of plant growth^51^. It was clear from the results that *B. subtilis* strain 330-2 is safe for the health of plants without any adverse effects. It is suggested that the *B. subtilis* strain 330-2 used in this study could be used as a PGPB^52^.

To assess the differential expression of genes, the most advanced and authentic R-PCR method was used to compare the *B. subtilis* strain 330-2 with *B. subtilis* 168. In total, 114 single clones were differentially expressed by R-PCR, suggesting the involvement of many genes in biotic and abiotic stress tolerance, as well as growth enhancement. Approximately 10% of fragments, including polyketide synthases (PKS) and non-ribosomal peptide synthetases (NRPSs), were involved in the synthesis of secondary metabolites and defense mechanisms for the PGPB-induced suppression of plant pathogens (Supplementary Table 2). Biosynthesis of bioactive lipopeptides plays an important role in the interaction of *Bacillus* species and plants either by suppressing the soil-borne pathogens in the rhizosphere or/and enhancing the plant defense mechanisms. Biosynthesis of bioactive lipopeptides occurs due to various non-ribosomal peptide synthetases (NRPSs) that have antagonistic activities^53^. Activation of several PKS/NRPS genes related to the production of antibiotics (e.g., *srfAA*) plays a key role in root colonization by emulsifying, foaming and decreasing the surface tension of solids to assure proper root colonization and a suitable environment that facilitates proliferation^54^. In a previous study, it has been reported that *srfAA*, macrolactin polyketide synthase, difficidin synthase and fengycin synthetase were induced in response to the root exudates and were directly linked to the biological control activity of *B. amyloliquefaciens* FZB42^55^. Bacteria in the rhizosphere sense the root exudates that are released by the plants via methyl-accepting proteins, which activates motility-related genes (e.g., *hag*, *mcpA*) and then move to the root surface for attachment^56^. In the present study, several genes associated with cell motility and biofilm formation (e.g., *epsB*, *wapA*, *spo0A*) were differentially expressed in *B. subtilis* strain 330-2. They are suspected to enhance the ability of *B. subtilis* strain 330-2 to exploit various plant-derived polysaccharides in the rhizosphere. These differentially expressed genes might favor host plant sensing and defense against pathogens in plants treated with *B. subtilis* strain 330-2. Iron has been reported to play a key role in bacterial biofilm formation^57^. In this study, the ferrichrome transport system permease protein (*fhuG*) gene was differentially expressed by strain 330-2 (Supplementary Table 2), which is known to be involved in scavenging iron from environmental sources^58^.

In the natural environment, *B. subtilis* is frequently faced with different stress factors, including salinity, oxidative stress, heat stress, and deprivation of nutrients^59^. These stress factors negatively influence the growth and productivity of living organisms, including plants and bacteria, leading to significant economic losses globally. Thus, it is useful to develop stress-tolerant species and to understand the underlying mechanisms^60^. Nine genes associated with abiotic stress tolerance (e.g., *yuaI*, *gpmI*, *glpT*, *coxA*, *glcD*, *yceD*, *yugJ*, *ggt*, *metK*) were differentially expressed in *B. subtilis* strain 330-2. Specifically, approximately 7, 5, 3, 2, and 1 genes related to tolerance against salinity, oxidative, heat, drought cold and oxidative stress, respectively, were differentially expressed in *B. subtilis* strain 330-2 (Fig. 8). These differentially expressed genes belonged to diverse functional classes, such as photosynthesis, lipid metabolism (G3P), stress/defense carbohydrate metabolism, competition and membrane transport (Supplementary Table 2). In the past, it has been reported that inoculation with PGPR enhanced the drought tolerance via inducing transcription of drought response genes^61^, affecting the phytohormonal balance^62^ and sugar accumulation^63^. Glycerol-3-phosphate (*glpT*), an important component of carbohydrates as well as lipid metabolic processes, enhanced the basal defense against *Colletotrichum higginsianum* ^64^. Likewise, the involvement of *yuaI*, and *gpmI* against salinity stress is evident^65, 66^. In the present study we found that the sporulation cortex protein (*coxA*) was differentially expressed in the strain 330-2, which is involved in low temperature stress^67^.

The genes differentially expressed by *B. subtilis* strain330-2 are involved in oxidative stress (Supplementary Table 2) responses and the activation of different cellular defense mechanisms. These genes primarily include glycolate oxidase (*glcD*), tellurium resistant (*yceD*), which is important for tellurite resistance^68^, putative NADPH-dependent butanol dehydrogenase (*yugJ*), which acts in direct detoxification of hydrogen peroxide^69^, and gamma-glutamyltranspeptidase (*ggt*), for resistance to organic peroxides^70^. Furthermore, we observed that *metK*, the precursor of ethylene and polyamine biosynthesis^71^, was differentially expressed by *B. subtilis* strain 330-2. This gene has been known to trigger tolerance against salinity^72^ and drought stresses^73^. In conclusion, we have presented strong evidence that using the R-PCR approach allowed the identification of a substantial number of resistant genes in biotic/abiotic stresses.

## Methods

### Strains and growth conditions

The *Bacillus subtilis* strain 330-2 used in this study was isolated from the rapeseed in Wuhan, China and maintained at −80 °C. The *Bacillus subtilis* strain 168 and phytopathogenic fungi, *Rhizoctonia solani* AG1-IA, *Fusarium oxysporum*, *Botrytis cinerea*, *Alternaria alternata*, *Cochliobolus heterostrophus*, and *Nigrospora oryzae*, were obtained from the Department of Plant Pathology, Huazhong Agricultural University, Wuhan, 430070, P.R. China. The bacterial strain *B. subtilis* strain 168 was initially streaked from −80 °C glycerol stocks onto LB medium plates and then was cultured from a fresh single colony into LB broth. The fungal cultures were maintained on potato dextrose agar (PDA) by sub-culturing at regular interval and incubated at 28 °C.

### Plant and soil sample collection

Samples of maize (*Zea mays* L.), wheat (*Triticum astevam* L.), rice (*Oryza sativa* L.), wild rapeseed (*Brassica napus*) and rhizosphere soils were collected from the field of Huazhong Agricultural University, Wuhan, 430070, P.R. China. Briefly, for endophytic isolation plant tissue samples were washed with running tap water to remove adherent soil particles and microbes. Samples were cut into 1–2 cm small pieces aseptically and surface sterilized with 70% ethanol for 1 min, 1.2% NaClO solution for 5 min, followed by 70% ethanol for 1 min. Then, samples were washed three times with sterilized distilled water and surface dried on sterilized filter paper. Surface sterilized samples were ground to a slurry with a sterilized mortar and pestle containing sterilized quartz sand. One gram each of rhizopheric soil, crushed roots, crushed leaves and crushed stem were homogenized separately in 9 ml saline (0.85% NaCl) in 20 ml test tube. The suspensions were homogenized, serial diluted up to 10^−5^. For each of these dilutions, 0.1 ml was spread on different Luria Bertani (LB) medium plates and then incubated at 28 °C for 4 days. The water from the last rinse was used as a control. Then, the colonies with different morphology were selected and purified by sub-culturing using maximum possible antiseptic condition^74^. Pure cultures were examined for growth studies.

### Isolation and identification of the bacterial strain

The bacterial *B*. subtilis strain 330-2 used in this experiment was isolated from wild rapeseed. *B. subtilis* strain 330-2 indicated the highest inhibitory effect *in vitro* against *R. solani* and other fungi. Phenotypic profiling of the *B. subtilis* strain 330-2 was tested using BIOLOG GenIII MicroPlates (Model EL311, BioTek Instruments, USA), containing 71 carbon source utilization assays and 23 chemical sensitivity assays according to the manufacturer’s protocol. The 10 μl microbial suspension was inoculated into each well of the GENIII MicroPlates. The plates were incubated at 30 °C for 24 to 48 h and observed for color development at 12 h intervals. Data were recorded at a 590 nm wavelength and the color development in the plates was read and compared in the BIOLOG database (Microlog SystemTm, Release 4.0) to identify the strain to species level^75^. The 16S rDNA gene of the strain was amplified using universal primer, both forward 27F (5′-AGAGTTTGATCCTGGCTCAG-3′) and reverse 1492R (5′-GGTTACCTTGTTACGACTT-3′)^76^. These primers were synthesized by T-Singke, Wuhan, China. The total volume (50 μl) of the PCR reaction mixture contained 5 μl of 10 × Taq buffer, 1.5 μl of MgCl_2_ (25 mmol L^−1^), 4 μl of each dNTPs (10 mmol L^−1^), 2 μl of each primer (30 pmol), 1 μl of DNA template, 0.25 μl of Taq DNA polymerase, and 35.75 μl of ddH_2_O. The PCR reaction was performed using MyCycler Thermal Cycler (Bio-RAD, Foster, California, USA) with the following thermal program: 95 °C for 5 min, followed by 94 °C for 30 s, 50 °C for 30 s, and 72 °C for 1 min 30 s. These steps were repeated for 30 cycles, and a final 10 min extension at 72 °C. The molecular size of the resulting PCR product was analyzed on a 1.0% agarose gel to confirm that the fragment was 1.5 kb, and the amplified 16S rDNA fragment was sequenced by T-Singke, Wuhan, China. The 16S rDNA sequence results were analyzed and compared on the website, http://www.ncbi.nlm.nih.gov ^77^.

### Indole-3-acetic acid (IAA) production detection

Detection of IAA production in the *B. subtilis* strain 330-2 and *B. subtilis* strain 168 were determined by the method reported by Brick *et al*.^78^. The strains were grown on LB broth and incubated at 28 °C for 48 h. Well-grown cultures were centrifuged at 10,000 rpm for 15 min at 4 °C to collect the supernatant. The supernatant (2 ml) was mixed with two drops of O-phosphoric acid and 4 ml of Salkowski reagent (50 ml, 35% of perchloric acid, 1 ml 0.5 M FeCl_3_ solution). The appearance of a pink color in the supernatant confirmed the production of IAA. After 30 min, the optical density (OD) of the mixture was read at an absorbance of 530 nm in a NanoDrop 2000 spectrophotometer (Thermo Fisher Scientific Inc., Wilmington, USA). The amount of IAA was estimated using a standard curve.

### Siderophore production detection

Siderophore production of the strains was determined on blue agar plates containing Chrome-azurol S (CAS) medium following Schwyn and Neilands’ method^79^. The bacterial strains (24 h-old culture) were spotted on CAS medium and incubated at 28 °C for 4 days. Formation of an orange halo around the colony was the indicator of siderophore production.

### Phosphate solubilization

Phosphate solubilization of the isolated strain 330-2 and *B. subtilis* strain 168 was detected by spotting these strains separately on Pikovskaya’s (PVK) agar plates using zinc phosphate (ZP), di-calcium phosphate (DCP), and tri-calcium phosphate (TCP) as sources of insoluble inorganic phosphate. Plates were incubated at 28 °C for 7 days^80^. The halo zone and colony diameter were measured and data were recorded. The phosphate solubilization index (S.I.) was calculated using the equation: S.I. = (colony diameter + halo zone diameter)/colony diameter^81^.

### Phytase production detection

Phytase production of the strains was evaluated by spot inoculation of log phase culture on phytase screening media using sodium and calcium phytate as sources of insoluble organic phosphate. The plates were incubated at 28 °C for 7 days. The halo zone around the colonies was observed on the plates.

### Zinc solubilization

To evaluate zinc solubilization abilities, the log phase culture of the bacterial strains was spotted on Tris-minimal medium plates containing zinc phosphate and zinc oxide as sources of insoluble inorganic zinc. The inoculated plates were then incubated at 28 °C for 7 days. Clearing zones around the colonies were observed for zinc solubilization.

### Cell wall degrading enzymes

β-1,3-glucanase and β-1,4-glucanase activities were studied using minimal medium (MM) containing laminarin azure (Sigma–Aldrich Co., USA) and cellulose powder as sole sources of carbon, respectively. Laminarin azure and cellulose amended minimal medium indicate the production of β-1,3-glucanase and β-1,4-glucanase, respectively. The medium plates were incubated at 28 °C for 7 days. Protease production was assessed by spotting each of the bacterial strains on Skim Milk Agar (SMA) medium plates. The bacterial strains that produced protease were identified by halo zones around the bacterial colonies^82^.

### *In vitro* antagonistic activity assays

The strain was tested for its ability to inhibit the growth of *R. solani* AG1-IA, *B. cinerea*, *F. oxysporum*, *A. alternata*, *C. heterostrophus*, and *N. oryzae in vitro*. The *B. subtilis* strain 330-2 was dabbed at two corners 3 cm from the center by stabbing the plate with sterilized tooth pick. After 24 h, fresh mycelium of test fungi (0.8-cm diameter) was placed in the center of the PDA plates. All treatments were conducted in triplicate. Plates were incubated at 28 °C. The inhibition of fungal growth was monitored by recording the diameter of the inhibition zone (mm).The percentage of inhibition was calculated using the following formula^83^.

$${\rm{Inhibition}}\,{\rm{rate}}\,( \% )=[({\rm{C}}-{\rm{T}})\times 100)/{\rm{C}}]$$

where “C” is the mycelium diameter (cm) of the fungus growing on the control plates (without bacteria) and “T” is the mycelium diameter (cm) of the fungus growing in the bacterial-treated plates. The experiment was repeated thrice with three biological replicates each time.

### Plant growth assay

*B. subtilis* strain 330-2 was grown in 100 ml LB medium in a rotary shaker (170 rpm) at 37 °C, until they reached an optical density at 600 nm (OD600) of 0.8. The bacterial cells were harvested by centrifugation at 6000 × *g* for 5 min at 4 °C and resuspended in 100 ml of distilled water and adjusted to (10^10^ CFU ml^−1^). Seeds of rice (*Oryza sativa*, L.) (variety: Huanghuazhan) and corn (*Zea mays* L.) (variety: B73) were obtained from the College of Plant Science and Technology of Huazhong Agricultural University, Wuhan, China. The seeds were surface sterilized with 70% alcohol for 1 min, followed by 0.5% NaClO solution for 1.30 min and 70% alcohol for 1 min. The seeds were then washed three times with distilled water. The sterilized seeds were bio-primed with bacterial culture for 4 h (control seeds were treated with distilled water). Seeds were then placed in the sterile petri dishes and dried back to their original moisture contents at room temperature. After bio-priming, seed germination tests were carried out. Ten rice seeds and three maize seeds for each treatment were placed in plastic pots of 7 cm diameter and 15 cm diameter, respectively and were filled with sterilized soil. Each treatment was replicated three times, respectively. Then, pots were incubated in a growth chamber at 28 °C with a photoperiod of 16 h light and 8 h dark. Data were calculated for shoot length, root length, fresh weight and dry weight.

### *B. subtilis* strain 330-2 effects in tobacco plants

The addition of the PGPB, which aimed to trigger plant growth, should not possess any detrimental effect on other plant species. *B. subtilis* strain 330-2 was tested in tobacco plants as a well-studied model and widely cultivated crop species^84^. Tobacco seeds were surface sterilized and grown in autoclaved soil in plastic pots (7 cm diameter). When the seedling shoots reached 1 cm, they were inoculated with a bacterial suspension (10^8^–10^9^ CFU ml^−1^). Four seedlings per condition were sampled on 7, 14, and 21 days, and data were recorded on plant height, fresh weight, and dry weight post inoculation. M9 buffer without bacterial inoculum was used as a negative control.

### Generation of differentially expressed clones by R-PCR

The removing PCR (R-PCR) is a restriction enzyme-based method, which eliminates unwanted genes from the gene population very efficiently. Using this method, the undesirable genes can be removed by means of removing drivers. This recently developed R-PCR technique was used to isolate the differentially expressed antagonism-related genes by comparing genomic DNA populations of the *B. subtilis* strain 330-2 and *B. subtilis* strain 168. To identify specific genes in *B. subtilis* strain 330-2, the *B. subtilis* strain 330-2 and *B. subtilis* strain 168 were taken as a tester and driver, respectively. The *B. subtilis* strain 168 is a well-known model strain without roles in enhancing plant growth and tolerance to biotic/abiotic stresses. The R-PCR was performed between the ‘driver’ and ‘tester’ according to the previous method of Huan *et al*.^33^. All PCR steps were performed on MyCycler Thermal Cycler (Bio-RAD, Foster, California, USA). Bands of the second R-PCR were excised from the gel, and PCR products were purified using the QIAquick gel purification kit (QIAGEN, Germany). The purified PCR products were cloned in pmD18-T Easy vector following manufacturer’s protocol (Promega) and transformed into DH5α cells. Plates were incubated at 37 °C until small colonies were visible. Sequencing of the positive clones were done by T-Singke, Wuhan, China using primers both forward M13F (−47) (5′-CGCCAGGGTTTTCCCAGTCACGAC-3′) and reverse M13R (−48) (5′-AGCGGATAACAATTTCACACAGGA-3′).

### Sequences analysis

A total of 180 white-clones were picked from the library. The inserted fragments were amplified, and fragments of the differentially expressed clones were sequenced using the primer pair of M13F and M13R. Sequences were trimmed of vector using DNAMAN software. Homology searches of all sequences were queried in the GenBank database with nucleotide blasts available at the NCBI (http://www.ncbi.nlm.nih.gov/BLAST).

### Statistical analysis

Data were analyzed by one way analysis of variance (ANOVA) using STATISTIX software (8.1) (Analytical Software, Tallahassee, FL, USA). The mean variance was analyzed using a least significant difference (LSD) test at 0.05 probability level.

## Electronic supplementary material

Supplementary Information
