# Supplementary material for: Isolated Bacillus subtilis strain 330-2 and its antagonistic genes identified by the removing PCR
Source: Sci Rep. 2017 May 11;7:1777. doi: 10.1038/s41598-017-01940-9 (PMC5431837; doi:10.1038/s41598-017-01940-9)
Supplement: Supplementary file 1 — Supplementary Information [file 41598_2017_1940_MOESM1_ESM.pdf]

## Supplementary Information

### Isolated *Bacillus subtilis* strain 330-2 and its antagonistic genes identified by the removing PCR

Zahoor Ahmad, Jia Wu, Lulu Chen and Wubei Dong*

**Supplementary Table 1:** Substrates utilized as carbon sources by *B. subtilis* strain 330-2 as determined by GENIII BIOLOG MicroPlates assay

| No. | Substrate | Reaction results |
|---|---|---|
| A1 | Negative Control | Negative Control |
| A2 | Dextrin | ± |
| A3 | D-Maltose | ± |
| A4 | D-Trehalose | ± |
| A5 | D-Cellobiose | ± |
| A6 | Gentiobiose | ± |
| A7 | Sucrose | ± |
| A8 | D-Turanose | ± |
| A9 | Stachyose | ± |
| A10 | Positive Control | Positive Control |
| A11 | pH 6 | + |
| A12 | pH 5 | + |
| B1 | D-Raffinose | ± |
| B2 | α-D-Lactose | ± |
| B3 | D-Melibiose | ± |
| B4 | β-Methyl-D-Glucoside | ± |
| B5 | D-Salicin | ± |
| B6 | N-Acetyl-D-Glucosamine | ± |
| B7 | N-Acetyl-β-D-Mannosamine | - |
| B8 | N-Acetyl-D-Galactosamine | - |
| B9 | N-Acetyl Neuraminic Acid | - |
| B10 | 1% NaCl | + |
| B11 | 4% NaCl | + |
| B12 | 8% NaCl | + |
| C1 | α-D-Glucose | + |
| C2 | D-Mannose | ± |
| C3 | D-Fructose | ± |
| C4 | D-Galactose | - |
| C5 | 3-Methyl Glucose | - |

| | | |
|---|---|---|
| C6 | D-Fucose | - |
| C7 | L-Fucose | - |
| C8 | L-Rhamnose | - |
| C9 | Inosine | - |
| C10 | 1% Sodium Lactate | + |
| C11 | Fusidic Acid | - |
| C12 | D-Serine | - |
| D1 | D-Sorbitol | + |
| D2 | D-Mannitol | ± |
| D3 | D-Arabitol | - |
| D4 | myo-Inositol | + |
| D5 | Glycerol | + |
| D6 | D-Glucose-6-PO4 | - |
| D7 | D-Fructose-6-PO4 | ± |
| D8 | D-Aspartic Aci | ± |
| D9 | D-Serine | - |
| D10 | Troleandomycin | - |
| D11 | Rifamycin SV | + |
| D12 | Minocycline | - |
| E1 | Gelatin | ± |
| E2 | Glycyl-L-Prolin | - |
| E3 | L-Alanine | ± |
| E4 | L-Arginine | ± |
| E5 | L-Aspartic Acid | + |
| E6 | L-Glutamic Acid | + |
| E7 | L-Histidine | ± |
| E8 | L-Pyroglutamic Acid | - |
| E9 | L-Serine | ± |
| E10 | Lincomycin | - |
| E11 | Guanidine HCl | ± |
| E12 | Niaproof 4 | - |
| F1 | Pectin | ± |
| F2 | D-Galacturonic Acid | ± |
| F3 | L-Galactonic Acid Lactone | + |
| F4 | D-Gluconic Acid | ± |
| F5 | D-Glucuronic Acid | ± |
| F6 | Glucuronamide | ± |
| F7 | Mucic Acid | - |
| F8 | Quinic Acid | - |
| F9 | D-Saccharic Acid | - |
| F10 | Vancomycin | - |
| F11 | Tetrazolium Violet | ± |
| F12 | Tetrazolium Blue | ± |
| G1 | p-Hydroxy Phenylacetic Acid | - |
| G2 | Methyl Pyruvate | ± |
| G3 | D-Lactic Acid Methyl Ester | - |
| G4 | L-Lactic Acid | ± |
| G5 | Citric Acid | + |
| G6 | α-Keto-Glutaric Acid | - |

| | | |
|---|---|---|
| G7 | D-Malic Acid | - |
| G8 | L-Malic Acid | + |
| G9 | Bromo-Succinic Acid | ± |
| G10 | Nalidixic Acid | - |
| G11 | Lithium Chloride | + |
| G12 | Potassium Tellurite | + |
| H1 | Tween 40 | - |
| H2 | γ-Amino-Butryric Acid | ± |
| H3 | α-Hydroxy Butyric Acid | - |
| H4 | β-Hydroxy-D，L Butyric Acid | ± |
| H5 | α-Keto-Butyric Acid | - |
| H6 | Acetoacetic Acid | - |
| H7 | Propionic Acid | - |
| H8 | Acetic Acid | - |
| H9 | Formic Acid | - |
| H10 | Aztreonam | ± |
| H11 | Sodium Butyrate | + |
| H12 | Sodium Bromate | - |

- negative reaction; + positive reaction; ± moderate

**Supplementary Table 2:** Comparison of R-PCR cloned up-regulated sequences involved in resistance with known related genes in literature

| S. No | Clone ID | Putative Function | Identities | involved in biotic/abiotic stress[a] | References |
|---|---|---|---|---|---|
| 1 | B-7 | Polyketide synthase type I Difficidin (*dfnF*) | 469/472 (99%) | Antibacterial activity | 85 |
| 2 | Q-10 | Macrolactin (*mlnF*) | 253/255 (99%) | Antibacterial production | 86 |
| 3 | M-1 | Bacillaene synthesis NRPS/PKS protein (*baeJ*) | 202/202 (100%) | Antibacterial Production | 87 |
| 4 | T-2 | Iturin A (*ituA*) | 275/275 (100%) | Antibiotic production | 88 |
| 5 | D-12 | Multidrug resistance ABC transporter ATP-binding (*bmrA*) | 120/120 (100%) | Antibiotic production | 89 |
| 6 | W-9 | Penicillin-binding protein 2B (*pbpB*) | 171/172 (99%) | Antibiotic resistance | 90 |
| 7 | S-6 | Alkaline phosphatase synthesis sensor protein (*phoR*) | 391/391 (100%) | Antifungal | 91 |

| | | | | | |
|---|---|---|---|---|---|
| 8 | H-7 | Fengycin synthetase (*fenA*) | 619/643 (97%) | Antifungal activity | 92, 93 |
| 9 | Q-3 | Beta-lactamase (*penP*) | 223224 (99%) | Antibiotic | 94, 95 |
| 10 | R-7 | Transport protein *tqsA* | 384/384 (100%) | Autoinducer-2 (AI-2) quorum sensor | 96 |
| 11 | G-12 | Putative tyrosine-protein kinase (*epsB*) | 314/314 (100%) | Biofilm formation | 97 |
| 12 | S-10 | Cell wall-associated protein (*wapA*) | 800/815 (99%) | Biofilm formation | 98,99 |
| 13 | C-5 | Stage 0 sporulation protein (*spo0A*) | 101/102 (99%) | Biofilm formation | 100, 101 |
| 14 | F-2 | Sporulation cortex protein (*coxA*) | 127/128 (99%) | Cold stress | 67 |
| 15 | N-6 | Chemotaxis protein (*mcpA*) | 201/201 (100%) | Colonization and competence | 56 |
| 16 | T-3 | Flagellin protein (*hag*) | 582/582 (100%) | Colonization and competence | 102 |
| 17 | E-14 | Sulfite reductase [NADPH] flavoprotein alpha-component (*cysJ*) | 218/220 (99%) | Detoxification | 103, 104 |
| 18 | A-2 | Cysteine desulfhydrase (*cysK*) | 325/327 (99%) | Drought stress | 105 |
| 19 | E-16 | Adenosylmethionine-8-amino-7-oxononanoate aminotransferase (*bioA*) | 326/327 (99%) | Drought stress | 106 |
| 20 | B-8 | Bacillibactin (*dhbF*) | 338/338 (100%) | Fungal and bacterial infections | 107, 108 |
| 21 | T-9 | Cell-division protein (*ftsH*) | 267/270 (99%) | Heat and drought stress | 109 |
| 22 | R-1 | Aspartokinase II (*lysC*) | 216/216 (100%) | Heat stress | 110 |
| 23 | R-9 | Dihydroorotase (*pyrC*) | 335/336 (99%) | Heat stress | 111 |
| 24 | X-2 | Elongation factor Tu (*tuf*) | 304/305 (99%) | Heat stress | 112 |
| 25 | E-6 | Phosphoenolpyruvate carboxykinase (*pckA*) | 369/369 (100%) | Nitrogen fixation | 113 |

| | | | | | |
|---|---|---|---|---|---|
| 26 | O-10 | N-acetylmuramoyl-L-alanine amidase (*cwlB*) | 293/295 (99%) | Motility | 114 |
| 27 | U-6 | Putative acyltransferase (*yfiQ*) | 255/255 (100%) | Oxidative and heat stress | 115 |
| 28 | P-5 | Tellurium resistance protein TerD2 (*yceD*) | 219/220 (99%) | Oxidative stress | 68 |
| 29 | T-10 | Gamma-glutamyltransferase (*ggt*) | 320/322 (99%) | Oxidative Stress | 70 |
| 30 | U-5 | Anthranilate synthase component 2 (*pabA*) | 311/314 (99%) | Oxidative stress and precursor of tryptophan | 116, 117 |
| 31 | U-9 | Glycolate oxidase, subunit (*glcD* ) | 224/225 (99%) | Oxidative stress | 68 |
| 32 | W-3 | Nitrate reductase subunit gamm (*narI*) | 152/152 (100%) | Oxidative stress | 118 |
| 33 | Q-1 | Phosphofructokinase | 291/291 (100%) | Oxygen deficiency stress. | 119 |
| 34 | N-5 | Virulence factor esxA (*yukE*) | 162/162 (100%) | Pathogenesis | 120 |
| 35 | P-6 | Putative capsular polysaccharide biosynthesis protein (*ywqC*) | 235/235 (100%) | Polysacharrides degridation | 121 |
| 36 | D-3 | Sn-glycerol-3-phosphate (*glpT*) | 140/140 (100%) | Salt and cold stress | 64, 122 |
| 37 | N-4 | Glutamate symporter (*gltT*) | 211/212 (99%) | Salt and drought stress | 123 |
| 38 | Q-6 | S-adenosylmethionine synthetase (*metK*) | 209/210 (99%) | Salt and drought stress | 71, 124 |
| 39 | O-2 | heat shock protein (*grpE*) | 237/237 (100%) | Salt and heat stress | 125 |
| 40 | N-9 | N-acetyltransferase (*yuaI*) | 193/196 (99%) | Salt and oxidative stress | 65 |
| 41 | A-20 | Cardiolipin synthetase (*ywiE*) | 281/283 (99%) | Salt stress | 126 |
| 42 | B-5 | Glutamate synthase (NADPH/NADH) (*gltA*) | 512/516 (99%) | Salt stress | 127 |
| 43 | C-18 | Glucosamine (*glmS*) | 346/354 (98%) | Salt stress | 128 |

| | | | | | |
|---|---|---|---|---|---|
| 44 | E-2 | Glutaminase-1 (*glsA1*) | 122/122 (100%) | Salt stress | 129 |
| 45 | G-3 | 4-hydroxy-3-methylbut-2-enyl diphosphate reductase (*ispH*) | 327/327 (100%) | Salt stress | 130 |
| 46 | M-8 | Phosphoglycerate mutase (*gpml*) | 250/250 (100%) | Salt Stress | 66, 131 |
| 47 | N-10 | Diaminopimelate epimerase (*dapF*) | 206/206 (100%) | Salt stress | 132 |
| 48 | E-10 | Alcohol dehydrogenase (*gbsB*) | 717/720 (99%) | Salt, heat and drought stress | 133, 134 |
| 49 | T-7 | Cystathionine beta-lyase (*metC*) | 380/389 (98%) | Salt, heat and drought stress | 135, 136 |
| 50 | F-8 | Membrane bound serine protease | 256/257 (99%) | Salt, heat and oxidative stress | 137 |
| 51 | T-6 | NADH-dependent butanol dehydrogenase A (*yugJ*) | 315/316 (99%) | Salt, heat and oxidative stress | 69, 138 |
| 52 | R-3 | formate dehydrogenase (*yjgC*) | 339/339 (100%) | Salt, heat, oxidative stress and Antibiotic stress | 139, 140, 141 |
| 53 | C-16 | Putative fluoride ion transporter (*crcB*) | 327/328 (99%) | Toxicity | 142 |
| 54 | A-1 | Ferrichrome transport (*fhu*) | 269/270 (99%) | Transport of hydroxamate siderophores | 58 |
| 55 | M-7 | 1,4-beta-xylanase (*xynA*) | 165/165 (100%) | Virulence | 143 |
| 56 | M-4 | two-component sensor histidine kinase | 260/262 (99%) | Virulence and Chemotaxis | 144 |
| 57 | X-7 | 2-methylcitrate dehydratase | 292/292 (100%) | | |
| 58 | O-3 | Uroporphyrin-III C-methyltransferase | 317/318 (99%) | | |
| 59 | N-1 | Hypothetical Protein | 264/266 (99%) | | |
| 60 | P-9 | Peptidase M42 family protein | 310/310 (100%) | | |
| 61 | A-3 | Hydroxyphenylacetat e | 627/627 (100%) | | |

| | | | |
|---|---|---|---|
| 62 | B-12 | DNA helicase II ATP-dependent DNA helicase (*pcrA*) | 266/269 (99%) |
| 63 | B-13 | Putative protein (*yshC*) | 241/241 (100%) |
| 64 | B-2 | Trihydroxycyclohexane | 310/311 (99%) |
| 65 | B-4 | Endochitinase | 241/243 (99%) |
| 66 | B-6 | Molybdenum ABC transporter | 340/341 (99%) |
| 67 | C-11 | transcriptional regulator | 364/364 (100%) |
| 68 | C-20 | Structural protein [Bacillus phage SPP1] | 273/286 (95%) |
| 69 | D-16 | Na+driven efflux transporter | 224/224 (100%) |
| 70 | D-19 | Cell division protein (*divIB*) | 321/324 (99%) |
| 71 | E-17 | Germination protein gerKB1 | 207/208 (99%) |
| 72 | E-8 | Glycyl-tRNA synthetase subunit beta | 372/372 (100%) |
| 73 | F-12 | Uncharacterized HTH (*ykvZ*) | 307/309 (99%) |
| 74 | F-14 | Uncharacterized protein (*ycnI*) | 331/331 (100%) |
| 75 | F-16 | Small acid-soluble spore protein B | 381/381 (100%) |
| 76 | F-19 | Chromosome partitioning protein SMC | 353/354 (99%) |
| 77 | F-20 | Methylitaconate isomerase | 317/318 (99%) |
| 78 | F-4 | Peptide ABC transporter | 351/355 (99%) |
| 79 | G-11 | L,D-transpeptidase | 350/350 (100%) |
| 80 | G-5 | Mrp-like Protein | 436/436 (100%) |
| 81 | H-10 | Prolyl-tRNA synthetase | 367/367 (100%) |
| 82 | H-8 | Succinyl-CoA3-ketoacid-CoA transferase | 71/89 (80%) |

| | | | |
|---|---|---|---|
| 83 | J-6 | Hypothetical protein | 447/448 (99%) |
| 84 | J-8 | Hypothetical protein | 234/234 (100%) |
| 85 | K-7 | Carbamoyl phosphate | 192/192 (100%) |
| 86 | K-9 | Transcroptional protien Hypo Pro | 182/182 (100%) |
| 87 | M-2 | Carboxy-7-deazaguanine synthase (*queE*) | 273/273 (100%) |
| 88 | M-6 | Glutamyl aminopeptidase | 188/188 (100%) |
| 89 | N-2 | Dihydroorotate dehydrogenase 1B | 282/284 (99%) |
| 90 | O-5 | Thymidine phosphorylase | 266/269 (99%) |
| 91 | P-1 | 50S ribosomal protein L10 | 171/171 (100%) |
| 92 | P-10 | P-type calcium transport ATPase | 295/295 (100%) |
| 93 | Q-2 | Molybdenum cofactor biosynthesis protein B | 150/151 (99%) |
| 94 | Q-5 | Laminin-like protein epi-1 | 266/268 (99%) |
| 95 | Q-7 | Chromosomal replication initiator protein (*dnaA*) | 187/187 (100%) |
| 96 | R-10 | Methionyl-tRNA formyltransferase (*fmt*) | 266/266 (100%) |
| 97 | R-5 | Peptide chain release factor RF-1 | 280/281 (99%) |
| 98 | S-1 | Major Facilitator Superfamily protein | 290/290(100%) |
| 99 | S-3 | PGA-synthesis regulator (*pgsR*) | 520/544 (96%) |
| 100 | S-7 | enterobactin esterase | 371/373 (99%) |
| 101 | S-9 | DNA binding protein | 356/393 (90%) |
| 102 | T-4 | BltR family transcriptional regulator | 297/299 (99%) |
| 103 | T-5 | Damage inducible ATP-dependent helicase | 254/254 (100%) |

| | | | |
|---|---|---|---|
| 104 | U-1 | Formyltetrahydrofolate deformylase | 277/278 (99%) |
| 105 | U-8 | Putative acylaminoacyl-peptidase (*yuxL*) | 277/277 (100%) |
| 106 | W-4 | Prophage-derived uncharacterized protein (*yopX*) | 231/247 (94%) |
| 107 | W-6 | Putative threonine-rich GPI-anchored glycoprotein | 232/236 (98%) |
| 108 | W-7 | Ribosomal RNA small subunit methyltransferase G | 252/253 (99%) |
| 109 | X-1 | NAD-dependent DNA ligase (*ligA*) | 277/279 (99%) |
| 110 | X-3 | Conserved protein of unknown function | 206/214 (96%) |
| 111 | X-4 | Catabolite control protein A | 240/242 (99%) |
| 112 | X-9 | Phage-like protein | 243/247 (98%) |
| 113 | Q-8 | Branched-chain amino acid transporter (*alzD*) | 308/310 (99%) |
| 114 | P-2 | Sugar kinase | 476/478 (99%) |

[a]: The number of identical amino acid residues/total number of residues included in sequence comparison analysis. Values in parenthesis are the identity scores as calculated by BLAST-n program in NCBI.

**References**


85. Chen, X. *et al*. Structural and Functional Characterization of Three Polyketide Synthase Gene Clusters in *Bacillus amyloliquefaciens* FZB 42. *J. Bacteriol.* **188**, 4024–4036; 10.1128/JB.00052-06 (2006).

86. He, F. *et al*. Genome sequence of the plant growth promoting strain *Bacillus amyloliquefaciens* subsp. plantarum B9601-Y2 and expression of mersacidin and other secondary metabolites. *J. Biotech.* **164**, 281–291; 10.1016/j.jbiotec.2012.12.014 (2013).

87. Müller, S. *et al*. Bacillaene and sporulation protect *Bacillus subtilis* from predation by *Myxococcus xanthus*. *Appl. Environ. Microbiol.* **80**, 5603-5610; 10.1128/AEM.01621-14 (2014).

88. Tsuge, K., Akiyama, T. & Shoda, M. Cloning, Sequencing, and Characterization of the Iturin A Operon. *J. Bacteriol.* **183**, 6265–6273; 10.1128/JB.183.21.6265-6273.2001 (2001).
89. Steinfels, E. et al. Characterization of YvcC (BmrA), a Multidrug ABC Transporter Constitutively Expressed in *Bacillus subtilis*. Biochem. **43**, 7491–7502; 10.1021/bi0362018 (2004).
90. Prigozhin, D. M., Krieger, I. V., Huizar, J. P., Mavrici, D. & Waldo, G. S. Sub family Specific Adaptations in the Structures of Two Penicillin-Binding Proteins from *Mycobacterium tuberculosis*. *PLOS ONE*. **9**, e116249; 10. 1371/journal.pone.0116249 (2014).
91. Guo, Q., Li, S., Lu, X., Li, B. & Ma, P. PhoR/PhoP two component regulatory system affects biocontrol capability of *Bacillus subtilis* NCD-2. *Genet. Mol. Biol.* **33**, 333–340; 10.1590/S1415-47572010005000032 (2010).
92. Dunlap, C. A., Michael, J., Bowman, B. & Schisler, D. A. Genomic analysis and secondary metabolite production in *Bacillus amyloliquefaciens* AS 43.3: A biocontrol antagonist of *Fusarium* head blight. *Bio. Control* **64**, 166–175; 10.1016/j.biocontrol.2012.11.002 (2013).
93. Zhang, C., Zhang, X. & Shen, S. Proteome analysis for antifungal effects of *Bacillus subtilis* KB-1122 on *Magnaporthe grisea* P131. *World J. Microbiol. Biotechnol.* **30**, 1763–1774; 10.1007/s11274-014-1596-1 (2014).
94. Fenselau, C. *et al*. Identification of β-Lactamase in Antibiotic-Resistant *Bacillus cereus* Spores. *Appl. Environ. Microbiol.* **74**, 904–906; 10.1128/AEM.00788-07 (2007).
95. Chen, Y., Succi, J., Tenover, F.C. & Koehler, T. M. Beta-lactamase genes of the penicillin-susceptible *Bacillus anthracis* Sterne strain. *J. Bacteriol.* **185**, 823-30; 10.1128/JB.185.3.823-830.2003 (2003).
96. Duanis, A. D., Steinberg, D., Chai, Y. & Shemesh, M. The LuxS Based Quorum Sensing Governs Lactose Induced Biofilm Formation by *Bacillus subtilis*. *Front. Microbiol.* **6**, 1517; 10.3389/fmicb.2015.01517 (2016).

97. Gerwig, J., Kiley, T. B., Gunka, K., Stanley, W. N. & Stulke, J. The protein tyrosine kinases EpsB and PtkA differentially affect biofilm formation in *Bacillus subtilis*. *Microbiol.* **160**, 682-691; 10.1099/mic.0.074971-0 (2014).
98. Zhu, L. *et al*. Functional characterization of cell wall associated protein WapA in *Streptococcus* mutans. *Microbiol.* **152**, 2395–2404; 10.1099/mic.0.28883-0 (2006).
99. Li, Y. *et al*. Live-cell and super-resolution imaging reveal that the distribution of wall-associated protein A is correlated with the cell chain integrity of *Streptococcus* mutans. *Mol. Oral. Microbiol.* **30**, 376-383; 10.1111/omi.12100 (2015).
100. Hamon, M. A. & Lazazzera, B. A. The sporulation transcription factor Spo0A is required for biofilm development in *Bacillus subtilis.* M. *Microbiol.* **42**, 1199–1209 (2001).
101. Beauregard, P. B., Chai, Y., Vlamakis, H., Losick, R. & Kolter. R. *Bacillus subtilis* biofilm induction by plant polysaccharides. *PNAS* E1621–E1630; 10.1073/pnas.1218984110 (2013).
102. Gao, S., Wu, H., Yu, X., Qian, L. & Gao, X. Swarming motility plays the major role in migration during tomato root colonization by *Bacillus subtilis* SWR01. *Biol. Con.* **98**, 11-17; 10.1016/j.biocontrol.2016.03.011 (2016).
103. Yarmolinsky, D., Brychkova, G., Fluhr, R. & Saqi, M. Sulfite reductase protects plants against sulfite toxicity. *Plant Physiol.* **161**, 725-743; 10.1104/pp.112.207712 (2013).
104. Kozmin, S. J., Wang, J. & Schaaper, R. M. Role for CysJ Flavin Reductase in Molybdenum Cofactor-Dependent Resistance of *Escherichia coli* to 6-N-Hydroxylaminopurine. *J. Bac.* **192**, 2026–2033; 10.1128/JB.01438-09 (2010).
105. Shi, H. *et al*. Hydrogen sulfide regulates abiotic stress tolerance and biotic stress resistance in *Arabidopsis*. *J. Int. Plant Biol.* **57**, 628–640; 10.1111/jipb.12302 (2015).
106. Sadder, M., Alsadon, A. & Wahb-Allah, M. Transcriptomic analysis of tomato lines reveals putative stress-specific biomarkers. *Turk J. Agric. Fores.* **38**, 700-715; 10.3906/tar-1312-17 (2013).

107. Dertz, E. A., Xu, J., Stintzi, A. & Raymond, K .N. Bacillibactin-Mediated Iron Transport in *Bacillus subtilis. J. Am. Chem. Soc.* **128**, 22–23; 10.1021/ja055898c (2006).
108. Li, B. *et al*. R. Responses of beneficial *Bacillus amyloliquefaciens* SQR9 to different soilborne fungal pathogens through the alteration of antifungal compounds production. *Front. Microbiol.* **5**, 636; 10.3389/fmicb.2014.00636 (2014).
109. Deuerling, E., Mogk, A., Richter, C., Purucker, M. & Schumann, W. The ftsH gene of *Bacillus subtilis* is involved in major cellular processes such as sporulation, stress adaptation and secretion. *Mol. Microbiol.* **23**, 921-933 (1997).
110. Stoveken, N. *et al*. Specialized Aspartokinase Enhances the Biosynthesis of the Osmoprotectants Ectoine and Hydroxyectoine in *Pseudomonas stutzeri* A1501. *J. Bac.* 4456–4468; 10.1128/JB.00345-11 (2011).
111. Assis-de-Gontijo. A. *et al*. The role of the de novo pyrimidine biosynthetic pathway in *Cryptococcus neoformans* high temperature growth and virulence. *Fungal Genet. Biol.* **70**, 12–23; 10.1016/j.fgb.2014.06.003 (2014).
112. Fu, J., Momcilovic, I. & Vara-Prasad, P. V. Roles of Protein Synthesis Elongation Factor EF-Tu in Heat Tolerance in Plants. *J. Bot.* **2012**, 835836; 10.1155/2012/835836 (2012).
113. Mulley, G. *et al*. Pyruvate is synthesized by two pathways in pea bacteroids with different efficiencies for nitrogen fixation. *J. Bac.* **192**, 4944–4953; 10.1128/JB.00294-10 (2010).
114. Lazarevic, V., Margot, P., Soldo, B. & Karamata, D. Sequencing and analysis of the *Bacillus subtilis* lytRABC divergon: a regulatory unit encompassing the structural genes of the Nacetylmuramoyl-L-alanine amidase and its modifier. *J. Gen. Microbiol.* **138**, 1949-1961; 10.1099/00221287-138-9-1949 (1992).
115. Ma, Q. & Wood, T. K. Protein Acetylation in Procaryotes Increases Stress Resistance. *Biochem. Biophys. Res. Commun.* **410**, 846–851; 10.1016/j.bbrc.2011.06.076 (2011).

116. Bodenhausen, N. & Reymond, P. Signaling pathways controlling induced resistance to insect herbivores in *Arabidopsis*. *Mol. Plant-Microbe Interac.* **20**, 1406-1420; 10.1094/MPMI-20-11-1406 (2007).
117. Zhang, W., Culley, D. E., Hogan, M., Vitiritti L. & Brockman, F. J. Oxidative stress and heat-shock responses in *Desulfovibrio vulgaris* by genome-wide transcriptomic analysis. *J. Microbiol.* **90**, 41–55; 10.1007/s10482-006-9059-9 (2006).
118. Mirleau, P., Philippot, L., Corberand, T. & Lemanceau, P. Involvement of Nitrate Reductase and Pyoverdine in Competitiveness of *Pseudomonas fluorescens* Strain C7R12 in Soil. *Appl. Environ. Microbiol.* **67**, 2627–2635; 10.1128/AEM.67.6.2627-2635.2001 (2001).
119. Mustroph, A. *et al*. Characterization of the phosphofructokinase gene family in rice and its expression under oxygen deficiency stress. *Front. Plant Sci.* **4**, 125; 10.3389/fpls.2013.00125 (2013).
120. Burts, M. L., Wade, A. W., DeBord, K. & Missiakas, D. M. EsxA and EsxB are secreted by an ESAT-6-like system that is required for the pathogenesis of *Staphylococcus aureus* infections. *PNAS* **102**, 1169–1174; 10.1073/pnas.0405620102 (2005).
121. Mijakovic, I. *et al*. Transmembrane modulator-dependent bacterial tyrosine kinase activates UDP-glucose dehydrogenase. *EMBO J*. **22**, 4709–4718; 10.1093/emboj/cdg458 (2003).
122. Hungria, M. *et al*. Tolerance to stress and environmental adaptability of *Chromobacterium violaceum*. *Genet. Mol. Res.* **3**, 102-116 (2004).
123. Zaprasis, A., Bleisteiner, M., Kerres, A., Hoffmann, T. & Bremer, E. Uptake of Amino Acids and Their Metabolic Conversion into the Compatible Solute Proline Confers Osmoprotection to *Bacillus subtilis*. *Appl. Environ. Microbiol.* **81**, 250–259; 10.1128/AEM.02797-14 (2015).
124. Espartero, J., Pintor-Toro, J. A. & Pardo, J. M. Differential accumulation of *S*-adenosylmethionine synthetase transcripts in response to salt stress. *Plant Mol. Biol.* **25**, 217–227; 10.1007/BF00023239 (1994).

125. López-Leal, G. *et al*. RNA-Seq analysis of the multipartite genome of *Rhizobium etli* CE3 shows different replicon contributions under heat and saline shock. *BMC Genomics* **15**, 770; 10.1186/1471-2164-15-770 (2014).
126. Tsai, M. *et al*. *Staphylococcus aureus* requires cardiolipin for survival under conditions of high salinity. *BMC Microbiol.* **11**, 13; 10.1186/1471-2180-11-13 (2011).
127. Saum, S. *et al*. Biochemical and Molecular Characterization of the Biosynthesis of Glutamine and Glutamate, Two Major Compatible Solutes in the Moderately Halophilic Bacterium *Halobacillus halophilus*. *J. Bac.* 6808–6815; 10.1128/JB.00781-06 (2006).
128. Kosová, K., Prášil, I. T., & Vítámvás, P. Protein Contribution to Plant Salinity Response and Tolerance Acquisition. *Int. J. Mol. Sci.* **14**, 6757–6789; 10.3390/ijms14046757, (2013).
129. Zhou, J., Zhou, J., Yang, H., Yan, C. & Huang, F. Characterization of a sodium-regulated glutaminase from cyanobacterium *Synechocystis* sp. PCC 6803. *CHINA SER. C.* **51**, 1066–1075; 10.1007/s11427-008-0137-2 (2008).
130. Ramos, A. A. *et al*. Molecular and functional characterization of a cDNA encoding 4-hydroxy-3-methylbut-2-enyl diphosphate reductase from *Dunaliella salina*. *J. Plant Physiol.* **166**, 968–977; 10.1016/j.jplph.2008.11.008 (2009).
131. Vermassen, A. *et al*. Adaptation of Staphylococcus xylosus to Nutrients and Osmotic Stress in a Salted Meat Model. *Front. Microbiol.* **7**, 87; 10.3389/fmicb.2016.00087 (2016).
132. Wang, H. *et al*. Identification of differentially expressed proteins of *Arthrospira* (Spirulina) plantensis-YZ under salt-stress conditions by proteomics and qRT-PCR analysis. *Proteome Sci.* **11**, 6; 10.1186/1477-5956-11-6 (2013).
133. Schroeter, R., Hoffmann, T., Voigt, B., Meyer, H. & Bleisteiner, M. Stress Responses of the Industrial Workhorse *Bacillus licheniformis* to Osmotic Challenges. *PLOS ONE* 8, e80956; 10.1371/journal.pone.0080956 (2013).
134. Jin, Y. Z. *et al*. The Alcohol Dehydrogenase Gene Family in Melon (*Cucumis melo* L.): Bioinformatic Analysis and Expression Patterns. *Front. Plant Sci.* **7**, 670; 10.3389/fpls.2016.00670 (2016).

135. Maimann, S. *et al*. Transgenic potato plants reveal the indispensable role of cystathionine β-lyase in plant growth and development. *Plant J.* **23**, 747–758; 10.1046/j.1365-313x.2000.00842.x (2000).

136. Gong, P. *et al*. Transcriptional profiles of drought-responsive genes in modulating transcription signal transduction, and biochemical pathways in tomato. *J. Exp. Bot.* **61**, 3563–3575; 10.1093/jxb/erq167 (2010).

137. Wonderling, L. D., Wilkinson, B. J. & Bayles, D. O. The *htrA* (*degP*) Gene of *Listeria monocytogenes* 10403S Is Essential for Optimal Growth under Stress Conditions. *Appl. Environ. Microbiol.* **70**, 1935–1943; 10.1128/AEM.70.4.1935-1943.2004 (2004).

138. Fernando, M. R., Nanri, H., Yoshitake, S., Nagata-Kuno, K. & Minakami, S. Thioredoxin regenerates proteins inactivated by oxidative stress in endothelial cells. *Eur. J. Biochem.* **209**, 917–922 (1992).

139. David, P. *et al*. Three highly similar formate dehydrogenase genes located in the vicinity of the B4 resistance gene cluster are differentially expressed under biotic and abiotic stresses in *Phaseolus vulgaris*. *Theor. Appl. Genet.* **121**, 87–103; 10.1007/s00122-010-1293-x (2010).

140. Ambard-Bretteville, F., Sorin, C., Rébeillé, F., Hourton-Cabassa, C. & des Francs-Small, C. C. Repression of formate dehydrogenase in *Solanum tuberosum* increases steady-state levels of formate and accelerates the accumulation of proline in response to osmotic stress. *Plant Mol. Bio.* **52**, 1153–1168; 10.1023/B:PLAN.0000004306.96945.ef (2003).

141. Helmann, J. *et al*. Global transcriptional response of *Bacillus subtilis* to heat shock. *J. Bac.* **183**, 7318–7328; 10.1128/JB.183.24.7318-7328.2001 (2001).

142. Baker, J. L. *et al*. Widespread Genetic Switches and Toxicity Resistance Proteins for Fluoride. *Science* **335**, 233–235; 10.1126/science.1215063 (2012).

143. Bibi, Z., Ansari, A., Zohra, R. R., Aman, A. & Qader, S. A. Production of xylan degrading endo-1, 4-β-xylanase from thermophilic *Geobacillus stearothermophilus* KIBGE-IB29. J. Rad. Res. Appl. Sci. **7**, 478–485; 10.1016/j.jrras.2014.08.001 (2014).

144. Swartz, T. E. *et al*. Blue-Light–Activated Histidine Kinases: Two-Component Sensors in Bacteria. *Science* **317**, 1090-1093; 10.1126/science.1144306 (2007).

**Supplementary Table 3:** R-PCR efficiency verified by sequence blast analysis against *B. subtilis* strain 168 libraries. Although sequenced genomic DNA fragments that having hits only in its own library are very likely good candidates, but *B. subtilis* strain 330-2 is non-sequenced so we analyzed the fragments by using MEGA5 version5.2 with *B. subtilis* strain 168, only DNA fragments having no hits in *B. subtilis* strain 168 library was used to evaluate R-PCR efficiency. [a]: hits of R-PCR cloned sequences in B.s168 library are substantial similar. [b]: hits of R-PCR cloned sequences in B.s168 library are substantial different, including three specific cases.

| Sequenced total DNA fragments | Repetitive Sequences | Single Tones | Unverified | Verified | Verified false[a] | Varied true[b] | R-PCR efficiency |
|---|---|---|---|---|---|---|---|
| 180 | 66 | 114 | 0 | 114 | 9 | 105 | 92.10% |

**Supplementary Table 4:** Verification of R-PCR cloned DNA fragments by comparison with published library sequences. Identities of R-PCR cloned genes between *Bacillus subtilis* strain 330-2 (B.s330-2) and of *Bacillus subtilis* strain 168 (B.s168) were pairwise analyzed. We compared the R-PCR cloned gene sequences of B.s330-2 by using MEGA5 version5.2 method, with B.s168 from National Center for Biotechnology and Information (NCBI). Identities of cloned genes between B.s330-2 and B.s168 were calculated by dividing identical bases by full length. [a]: mutations of *Mse*I and *Apek*I recognition sites or partial sequence absence are labeled. [b]: if identity is less than 87% or at least one restriction recognition site is mutated/partial sequence is missing, it is taken as true for the R-PCR result; otherwise, it is false.

| Sequence No | Clone ID | Identical bases (bp) | Total length (bp) | Identities | Restriction recognition sites [a] | Verified true or false [b] |
|---|---|---|---|---|---|---|
| 1 | K-7 | 192 | 192 | 100 | | F |
| 2 | X-2 | 180 | 184 | 97.83 | Mutated | T |
| 3 | W-3 | 143 | 152 | 94.08 | Mutated | T |
| 4 | W-7 | 234 | 253 | 92.49 | | F |
| 5 | P-1 | 158 | 171 | 92.4 | | F |
| 6 | Q-3 | 200 | 224 | 89.29 | Mutated | T |
| 7 | O-3 | 281 | 318 | 88.36 | | F |
| 8 | O-2 | 208 | 237 | 87.76 | Mutated | T |

| | | | | | | |
|---|---|---|---|---|---|---|
| 9 | H-8 | 78 | 89 | 87.64 | | F |
| 10 | B-2 | 272 | 311 | 87.46 | | F |
| 11 | B-12 | 235 | 269 | 87.36 | | F |
| 12 | S-9 | 343 | 393 | 87.28 | | F |
| 13 | H-10 | 320 | 367 | 87.19 | | F |
| 14 | S-10 | 709 | 815 | 86.99 | Mutated | T |
| 15 | T-9 | 234 | 270 | 86.67 | Mutated | T |
| 16 | T-3 | 480 | 582 | 82.47 | Mutated | T |
| 17 | U-1 | 226 | 278 | 81.29 | | T |
| 18 | R-9 | 268 | 336 | 79.76 | Mutated | T |
| 19 | X-4 | 193 | 242 | 79.75 | | T |
| 20 | U-9 | 179 | 225 | 79.56 | | T |
| 21 | D-16 | 177 | 224 | 79.02 | | T |
| 22 | F-16 | 300 | 381 | 78.74 | | T |
| 23 | N-10 | 160 | 206 | 77.67 | Mutated | T |
| 24 | C-5 | 79 | 102 | 77.45 | | T |
| 25 | A-20 | 218 | 283 | 77.03 | | T |
| 26 | F-19 | 271 | 354 | 76.55 | | T |
| 27 | E-8 | 284 | 372 | 76.34 | | T |
| 28 | M-1 | 153 | 202 | 75.74 | | T |
| 29 | W-9 | 130 | 172 | 75.58 | | T |
| 30 | G-11 | 261 | 350 | 74.57 | | T |
| 31 | M-2 | 199 | 273 | 72.89 | | T |
| 32 | M-8 | 181 | 250 | 72.4 | Mutated | T |
| 33 | G-12 | 227 | 314 | 72.29 | Mutated | T |
| 34 | S-7 | 268 | 373 | 71.85 | | T |
| 35 | R-10 | 191 | 266 | 71.8 | | T |
| 36 | H-7 | 457 | 643 | 71.07 | | T |
| 37 | C-16 | 230 | 328 | 70.12 | Mutated | T |
| 38 | E-16 | 229 | 327 | 70.03 | Mutated | T |
| 39 | F-12 | 212 | 309 | 68.61 | | T |
| 40 | P-10 | 197 | 295 | 66.78 | | T |
| 41 | E-2 | 81 | 122 | 66.39 | | T |
| 42 | O-10 | 194 | 295 | 65.76 | | T |
| 43 | A-3 | 401 | 627 | 63.96 | | T |
| 44 | R-3 | 216 | 339 | 63.72 | | T |
| 45 | E-14 | 201 | 320 | 62.81 | | T |
| 46 | P-6 | 147 | 235 | 62.55 | | T |
| 47 | N-1 | 166 | 266 | 62.41 | | T |
| 48 | R-5 | 175 | 281 | 62.28 | | T |

| | | | | | |
|---|---|---|---|---|---|
| 49 | E-10 | 444 | 720 | 61.67 | T |
| 50 | T-10 | 194 | 322 | 60.25 | T |
| 51 | A-2 | 197 | 327 | 60.24 | T |
| 52 | Q-2 | 90 | 151 | 59.6 | T |
| 53 | M-7 | 98 | 165 | 59.39 | T |
| 54 | N-4 | 125 | 212 | 58.96 | T |
| 55 | B-7 | 275 | 472 | 58.26 | T |
| 56 | Q-7 | 108 | 187 | 57.75 | T |
| 57 | M-4 | 150 | 262 | 57.25 | T |
| 58 | T-2 | 157 | 275 | 57.09 | T |
| 59 | D-3 | 79 | 140 | 56.43 | T |
| 60 | K-9 | 102 | 182 | 56.04 | T |
| 61 | F-2 | 71 | 128 | 55.47 | T |
| 62 | Q-6 | 116 | 210 | 55.24 | T |
| 63 | B-13 | 133 | 241 | 55.19 | T |
| 64 | D-12 | 65 | 120 | 54.17 | T |
| 65 | N-9 | 105 | 196 | 53.57 | T |
| 66 | C-20 | 153 | 286 | 53.5 | T |
| 67 | T-7 | 208 | 389 | 53.47 | T |
| 68 | X-9 | 132 | 247 | 53.44 | T |
| 69 | J-8 | 125 | 234 | 53.42 | T |
| 70 | Q-5 | 143 | 268 | 53.36 | T |
| 71 | P-5 | 117 | 220 | 53.18 | T |
| 72 | R-7 | 204 | 385 | 52.99 | T |
| 73 | F-8 | 136 | 257 | 52.92 | T |
| 74 | F-20 | 168 | 318 | 52.83 | T |
| 75 | P-2 | 252 | 478 | 52.72 | T |
| 76 | A-1 | 142 | 270 | 52.59 | T |
| 77 | T-4 | 157 | 299 | 52.51 | T |
| 78 | R-1 | 113 | 216 | 52.31 | T |
| 79 | N-6 | 104 | 201 | 51.74 | T |
| 80 | Q-1 | 150 | 291 | 51.55 | T |
| 81 | C-11 | 187 | 364 | 51.37 | T |
| 82 | O-5 | 138 | 269 | 51.3 | T |
| 83 | X-1 | 142 | 277 | 51.26 | T |
| 84 | B-8 | 173 | 338 | 51.18 | T |
| 85 | B-5 | 264 | 516 | 51.16 | T |
| 86 | X-3 | 109 | 214 | 50.93 | T |
| 87 | S-3 | 277 | 544 | 50.92 | T |
| 88 | F-14 | 168 | 331 | 50.76 | T |

| | | | | | |
|---|---|---|---|---|---|
| 89 | N-5 | 82 | 162 | 50.62 | T |
| 90 | U-6 | 129 | 255 | 50.59 | T |
| 91 | M-6 | 95 | 188 | 50.53 | T |
| 92 | T-5 | 128 | 254 | 50.39 | T |
| 93 | Q-8 | 155 | 310 | 50 | T |
| 94 | B-6 | 170 | 341 | 49.85 | T |
| 95 | W-4 | 134 | 270 | 49.63 | T |
| 96 | D-19 | 160 | 324 | 49.38 | T |
| 97 | G-3 | 117 | 237 | 49.37 | T |
| 98 | P-9 | 153 | 310 | 49.35 | T |
| 99 | J-6 | 220 | 448 | 49.11 | T |
| 100 | U-5 | 153 | 314 | 48.73 | T |
| 101 | X-7 | 142 | 292 | 48.63 | T |
| 102 | N-2 | 138 | 284 | 48.59 | T |
| 103 | G-5 | 211 | 436 | 48.39 | T |
| 104 | B-4 | 117 | 243 | 48.15 | T |
| 105 | T-6 | 152 | 316 | 48.1 | T |
| 106 | F-4 | 170 | 355 | 47.89 | T |
| 107 | E-6 | 176 | 369 | 47.7 | T |
| 108 | E-17 | 97 | 208 | 46.63 | T |
| 109 | S-6 | 182 | 391 | 46.55 | T |
| 110 | C-18 | 161 | 354 | 45.48 | T |
| 111 | W-6 | 105 | 236 | 44.49 | T |
| 112 | Q-10 | 110 | 255 | 43.14 | T |
| 113 | S-1 | 125 | 290 | 43.1 | T |
| 114 | U-8 | 97 | 277 | 35.02 | T |
| | Average | 184.98 | 295.25 | 62.80 | |

**Supplementary Figure 1:** Biosafety and plant growth promotion by bacterial strain. Plant shoot length, plant fresh and dry weight in uninoculated tobacco plants and plants inoculated with *B. subtilis* strain 330-2. The values shown are the mean and standard deviation of four measurements. Small alphabetical letters (a, b...) above mean bars show the differences (P<0.05) among treatments within a specific parameters.

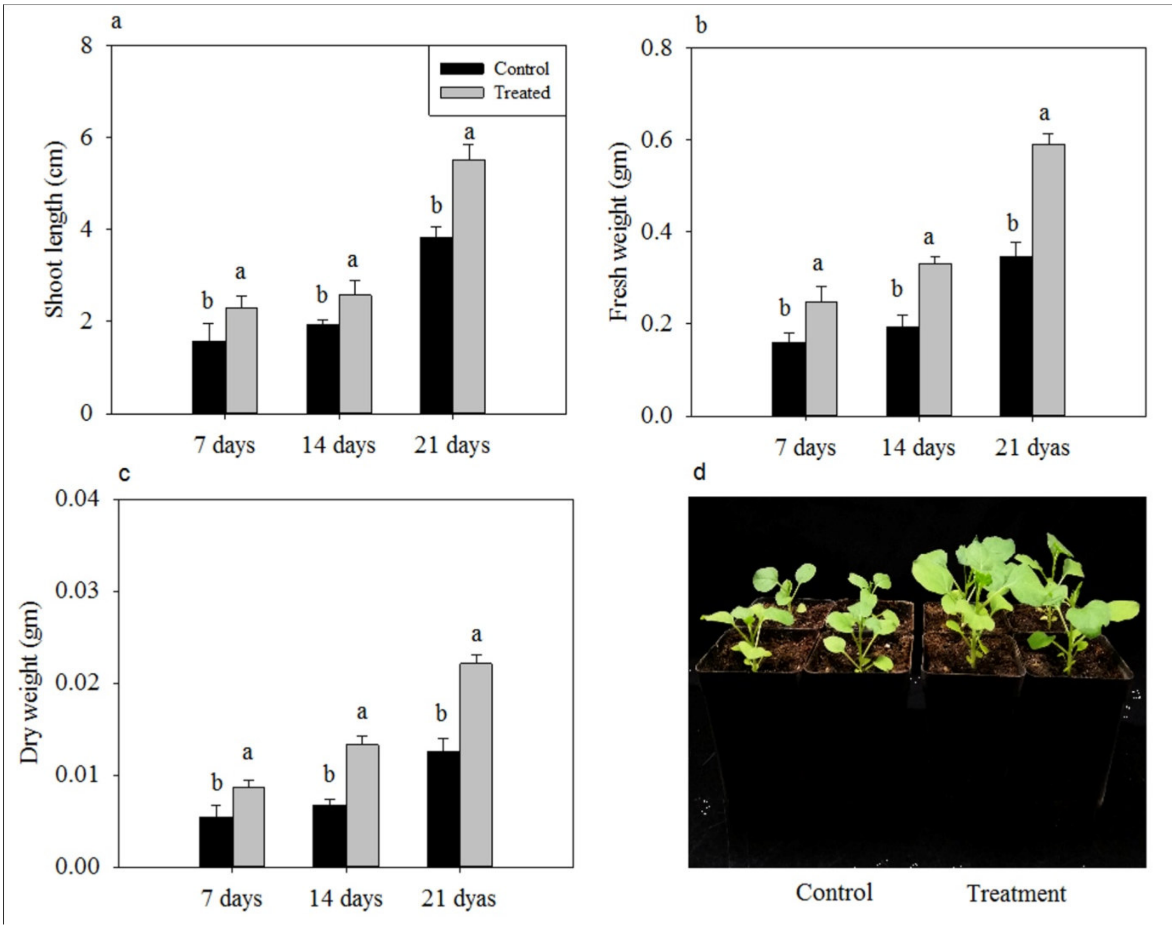

**Supplementary Figure 2:** Sequence distribution of annotated fragments involved in a) Biological processes, b) Molecular function, and c) Cellular components

a) Biological processes

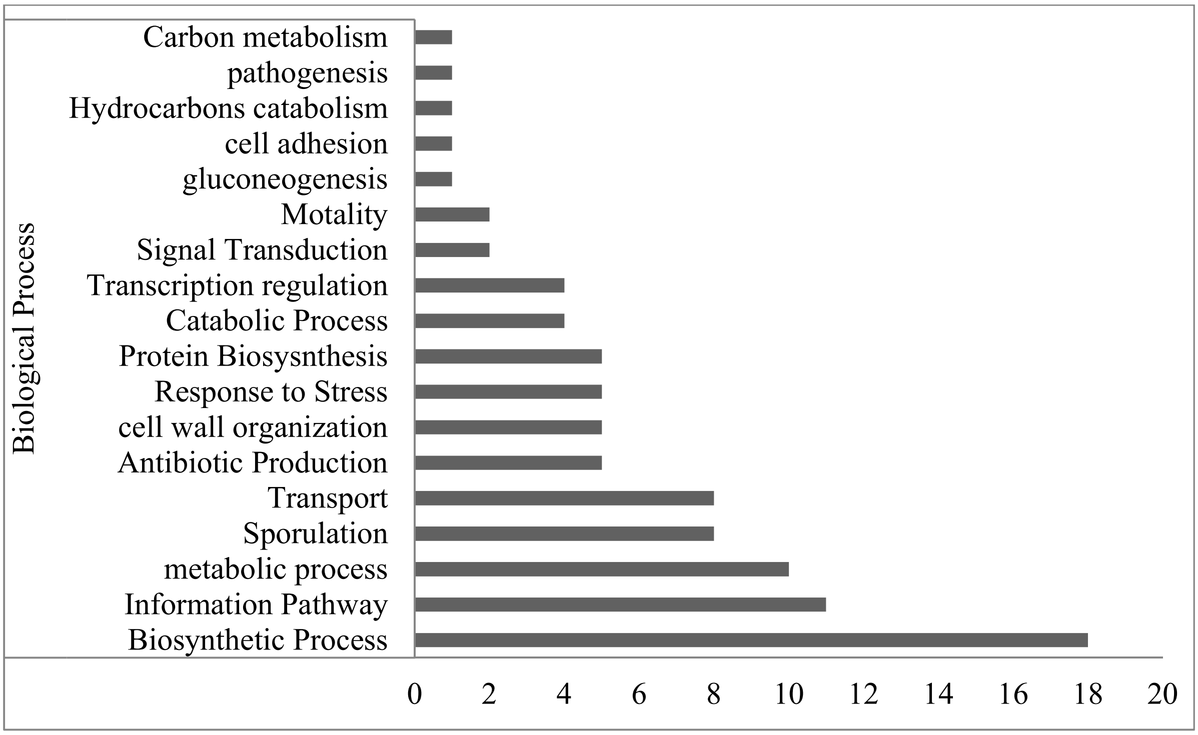

b) Molecular function

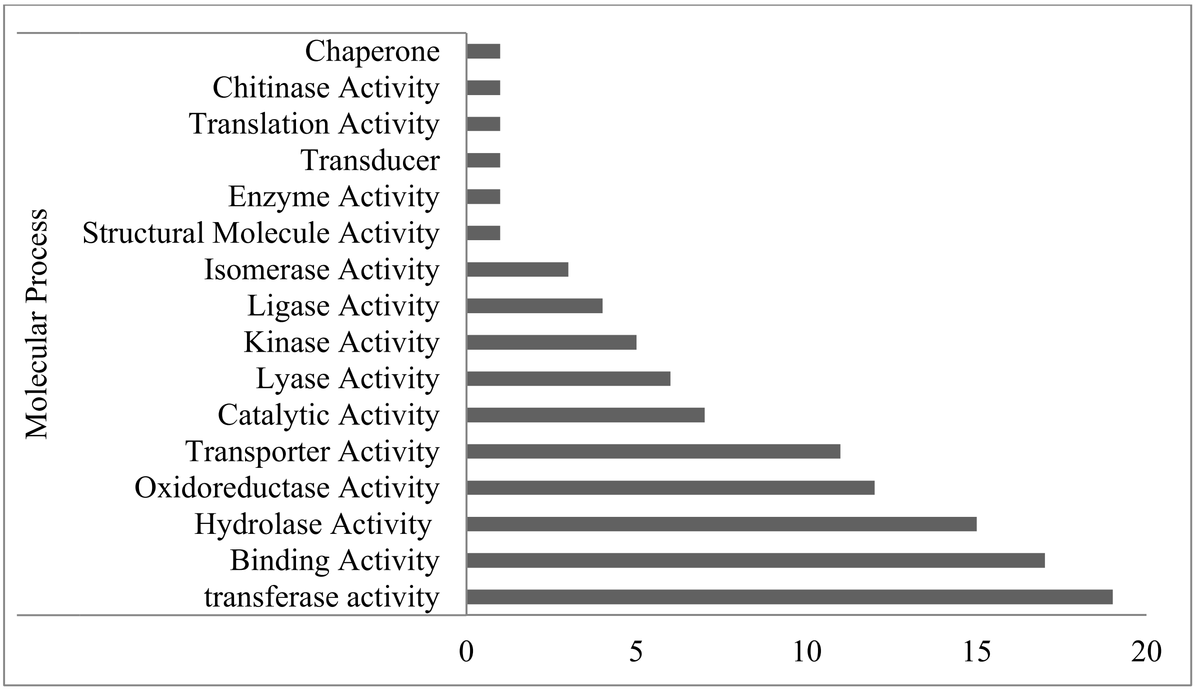


c) Cellular components

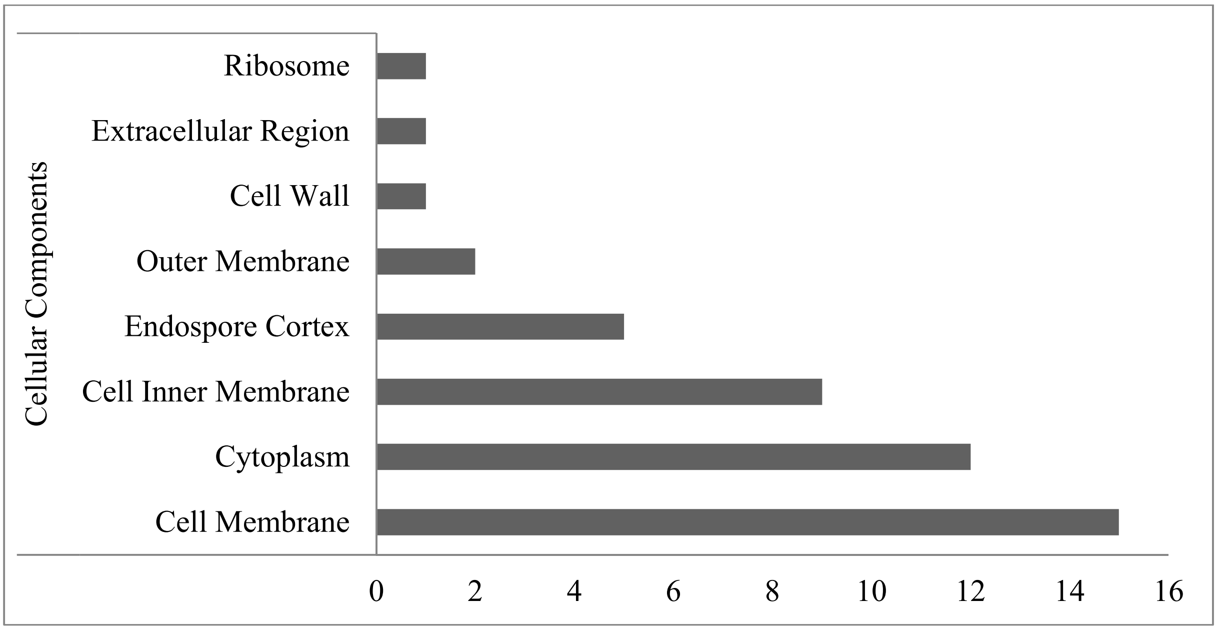

**Supplementary Figure 3:** Sequence comparisons of selected tester and driver. With three main differences, the driver could not eliminate the tester in R-PCR reactions. Tester was made from *B. subtilis* strain 330-2 (B.s330-2) and driver was made from *B. subtilis* strain 168 (B.s168). The recognition cutting sites of the *Mse*I and *ApeK*I (in black boxes) have mutations. *: Identical.

1. Ferrichrome transport (*fhuG*)

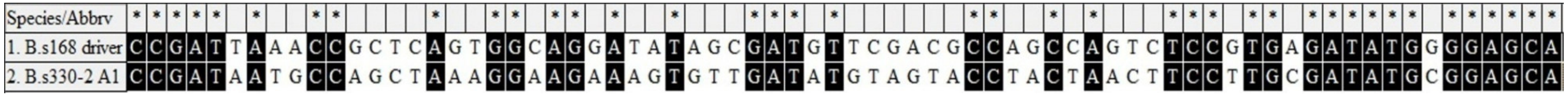


2. Cysteine desulfhydrase (*cysK*)

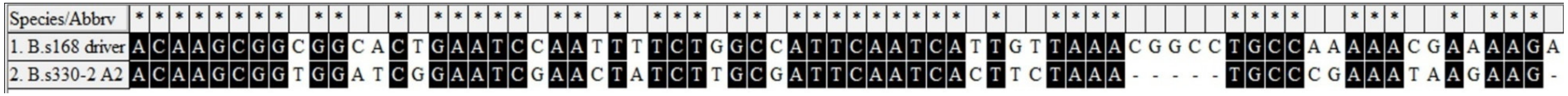


3. Cardiolipin synthetase (*ywiE*)

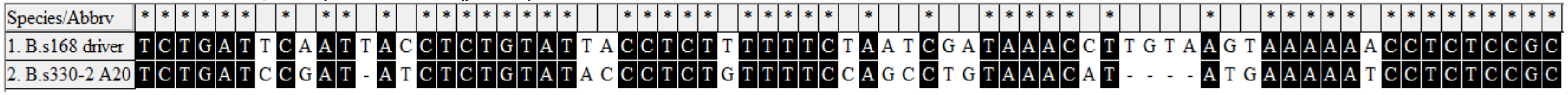


4. Glutamate synthase (NADPH/NADH) (*gltA*)

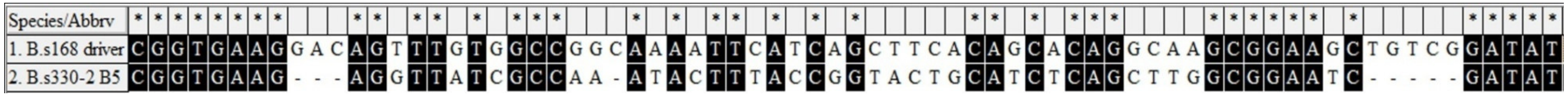


5. Polyketide synthase type I Difficidin (*dfnF*)

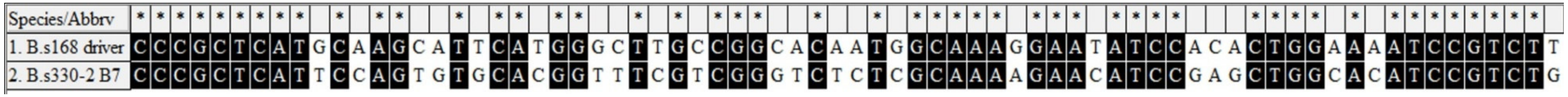

6. Bacillibactin (*dhbF*)

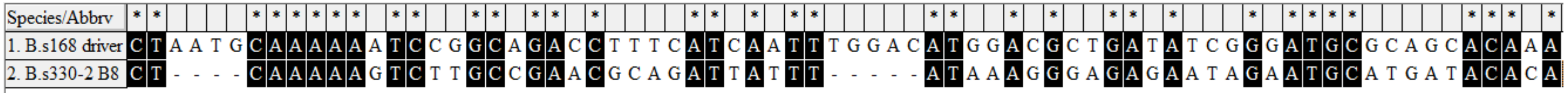


7. Stage 0 sporulation protein (*spo0A*)

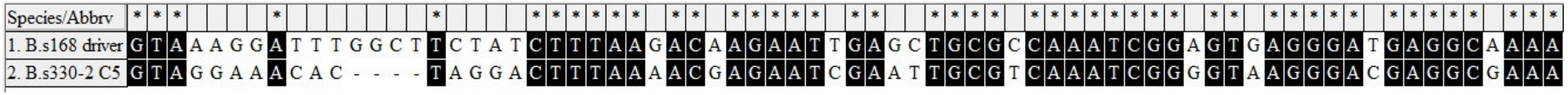


8. Putative fluoride ion transporter (*crcB*)

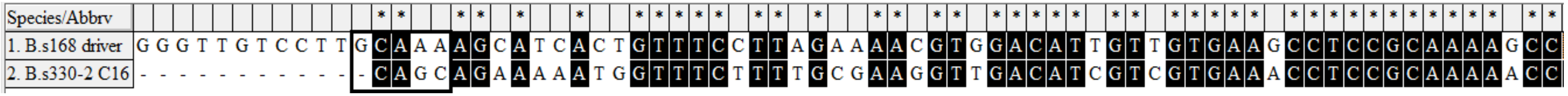


9. Glucosamine (*glmS*)

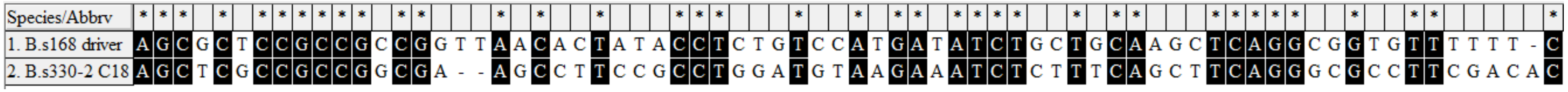


10. Sn-glycerol-3-phosphate (*glpT*)

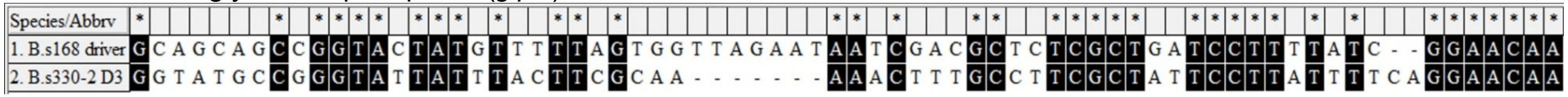


11. Multidrug resistance ABC transporter ATP-binding (*BmrA*)

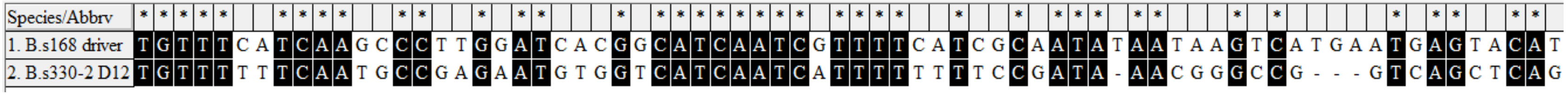

12. Glutaminase-1 (*glsA1*)

| Species/Abbrv | ********** ***********    **  ***  **   *   *****  * ** ** ******** **** |
| --- | --- |
| 1. B.s168 driver | TGACGTGCGGAATGTACAATGCAAGCGGGGAATTCGCAATTAAGGTCGGAATTCCTGCGAAAAGCGGCGTAT |
| 2. B.s330-2 E2 | TGACGTGCGGGATGTACAATGCGTCAGGCAAATATGCGGCTTTCGTCGGCGTCCCGGCCAAAAGCGGGGTAT |

13. Phosphoenolpyruvate carboxykinase (*pckA*)

| Species/Abbrv | ***** ***   *  *** ***   * * * * *    **** ***  **      * ** * *    * ** |
| --- | --- |
| 1. B.s168 driver | CAATCAGATCGCCTACTCCTGTCAATCCAGAGAACGTCAAGGGATTTCCGCCCATTTTCGTTCCGAGTCTCG |
| 2. B.s330-2 E6 | CAATCGGATATGCGGCTCTTGTGTTTTCCGTGTA---GAAGGAATTGTCGTAA---TCCGCTTCA---CCCG |

14. Alcohol dehydrogenase (*gbsB*)

| Species/Abbrv | ****      ***    * ***   *  **   *  **      ** ** *    *** * ***** *  ** |
| --- | --- |
| 1. B.s168 driver | CGTTTGGTGACAATGGAAGTGAGTCCAGTTTTTCAATAAACGCAGGGTGCATAAACACCGGCAGCTTGTTTT |
| 2. B.s330-2 E10 | CGTTC--CCGCAAGCATAATGA--TCGTTCCGCCGTAT-----GGATGAACGG-CACGGACAGCTCGCCTT |

15. Sulfite reductase [NADPH] flavoprotein alpha-component (*cysJ*)

| Species/Abbrv | ***** ** ******** **   ************ ******** ** ***** ******** ****  ** |
| --- | --- |
| 1. B.s168 driver | TGCACTTCATCCGGATTTGCGGATAAACTGCTTGCAATCGAATAAAGGCGGGCAGGCATTTTTCGCAAAATC |
| 2. B.s330-2 E14 | TGCACCTCTTCCGGATTCGCTTCTAAACTGCTTGCGATCGAATAGAGCCGGGCCGGCATTTTACGCAGGATG |

16. Adenosylmethionine-8-amino-7-oxononanoate aminotransferase (*bioA*)

| Species/Abbrv | *********   ******** ** ** ***** ** * *** ** *********** |
| --- | --- |
| 1. B.s168 driver | ACAAGCTCTGCACCGCACATAAAGCCAAGCTGCCGAATATCCCCAACATGAGGAAGAGCGTGCAGATCTTGA |
| 2. B.s330-2 E16 | ACAAGCTCTATGCCGCACATCAAACCGAGCTGGCGGACATCGCCGACATGAGGAAGCAG------------- |

17. Sporulation cortex protein (*coxA*)

| Species/Abbrv | **** *   * *  * ******** * ***    **     ** ***    ** *  *  * * **   * |
| --- | --- |
| 1. B.s168 driver | TTTTGCTGGAGAACAGGATAAACA-GCCAAGTGTATAAAAGCATAAGCCCCGCAGCTGTTGTCATATGCTCA |
| 2. B.s330-2 F2 | TTTTACGAAAAATGATGATAAACATGTCAAAC--ATGCGGCAAAAGATTTGCGGACG--GCCGTACAATT- |

### 18. Membrane bound serine protease

| Species/Abbrv | ****** *  *        * *  *   * *   * *** *  **    ***** * **** ** *    ** |
|---|---|
| 1. B.s168 driver | TCGGCGCAAAGCATGCAAACAGGAGCAGCACAGACAGCACCCGTATAAGCATTTGCACCGCCTGAATGGAAC |
| 2. B.s330-2 F8 | TCGGCGGAGTG---ATCGGCGGAGGGCTCGCCCTCGGCATCTATA----CATTTACGCCGCTTGGA---GAC |

### 19. 4-hydroxy-3-methylbut-2-enyl diphosphate reductase (*ispH*)

| Species/Abbrv | *** ** *****    *  *        * ***  *   ** *   ** *  ** *  *   * * * ** |
|---|---|
| 1. B.s168 driver | AAA-CGAATGACCAAATGGGGGGCACTCGCCGGCATGATTGTGGGAGCAGCGACTGTAATCATTTGGGCGAA |
| 2. B.s330-2 G3 | AAAGCGGATGACTTCCTTTGTAATCGGTGTCGGTGTTGATGCGCCTGCGGTAACGGCGACGGTATTGACGTC |

### 20. Putative tyrosine-protein kinase (*epsB*)

| Species/Abbrv | ***   **    *****    *****  * *** * *********** |
|---|---|
| 1. B.s168 driver | TCAGGAACGGAGCCTAAGCATGGCAATCCGGTTCTCTCGCTGAGCTGCCGTGCGCTTTTGCATGTATCATCG |
| 2. B.s330-2 G12 | TCATACACAACCCCTAAAACAGGCAAGTCTGTTTTTTCGCTGAGCTG------------------------- |

### 21. Fengycin synthetase (*fenA*)

| Species/Abbrv | ** **  *     *   * ***  * ******  **  **    *          *** *  ***   ***** |
|---|---|
| 1. B.s168 driver | CGGCTCCTGGAGATCCGCAAGCTTCAGCAAGATCAACAGCAGCTTCTGTATGGCCCGCTCTTTTCAGGACAC |
| 2. B.s330-2 H7 | CGACTTTT-----TTTCCGAGCCGC-GCAAGAGAAATGGCAGGTTGACGGAAACCGTTGATTTGCCGACAC |

### 22. Bacillaene synthesis NRPS/PKS protein (*baeJ*)

| Species/Abbrv | ************** **  ******* ****** * ** *  * ** *  *    * ******     ** |
|---|---|
| 1. B.s168 driver | CCGATTGAAATAAATGGGCTGAAAGCCGCGTTTAAGGAACTATCCAATATGAGAGGCGAGAGCCAGCCGGAT |
| 2. B.s330-2 M1 | CCGATTGAAATAAACGGTTTGAAAGCGGCGTTTCACGAGCTGGCGAAAACAAA--TCAAGAGCCT----GAA |

### 23. Two-component sensor histidine kinase

| Species/Abbrv | ******** ** * *    * * *  ** *   ***  *  *  * ****   ***   **    **  *** |
|---|---|
| 1. B.s168 driver | CCGTGTAATAAGGGTCAAACACCCGCTTGATGTCTTGTGAGGGAATCCCGACACCGTAATCTTTGACTTCAA |
| 2. B.s330-2 M4 | CCGTGTAAAAAAGCTGTGTCGCTCTTTTCAGCGCTTCCGGCGAGAACCCGCTTCCGCTGTCGGATACGGCAA |

24. Phosphoglycerate mutase (*gpmI*)

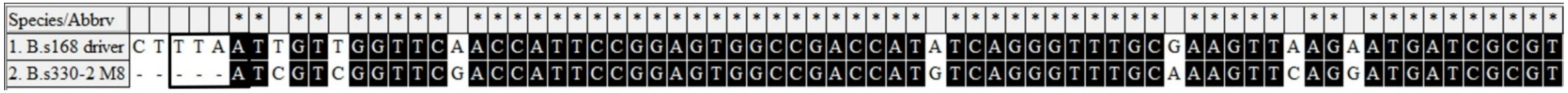


25. 1,4-beta-xylanase (*xynA*)

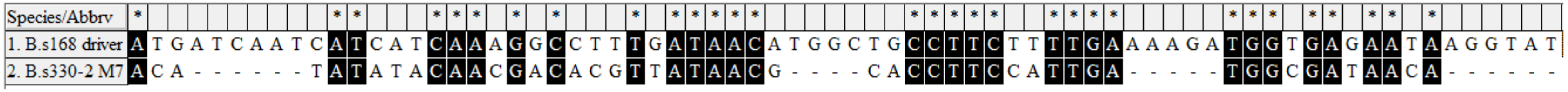


26. Glutamate symporter (*gltT*)

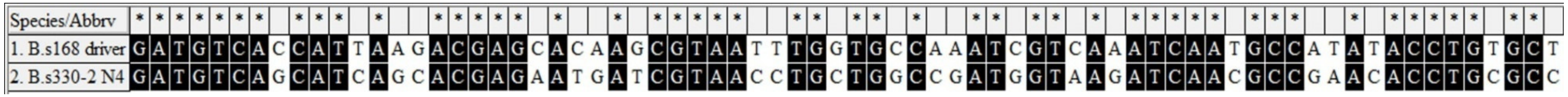


27. Virulence factor esxA (*yukE*)

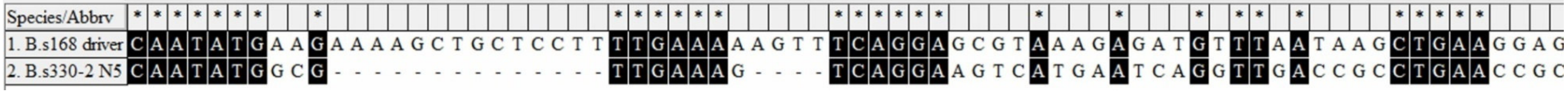


28. Chemotaxis protein (*mcpA*)

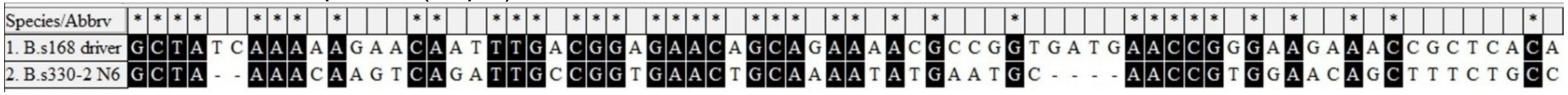


29. N-acetyltransferase (*yuaI*)

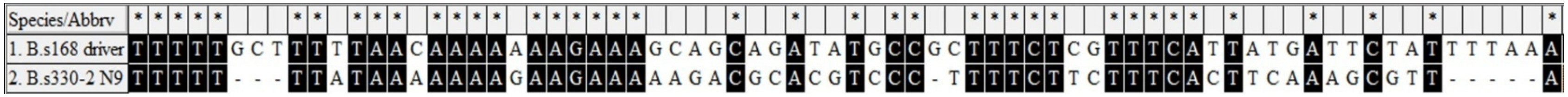

30. Diaminopimelate epimerase (*dapF*)

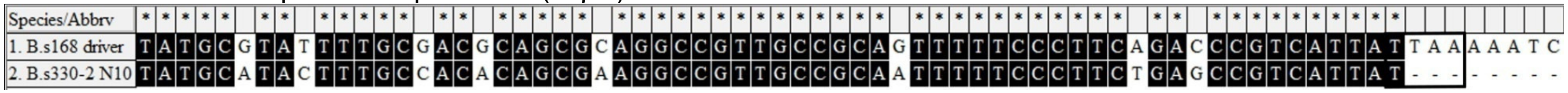


31. Heat shock protein (*grpE*)

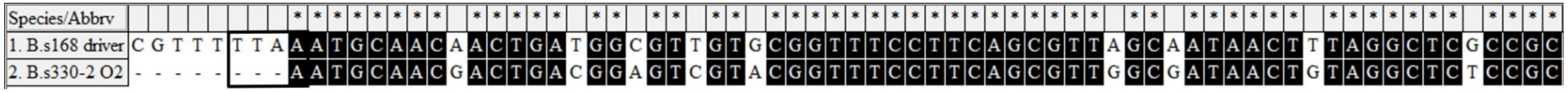


32. Tellurium resistance protein TerD2 (*yceD*)

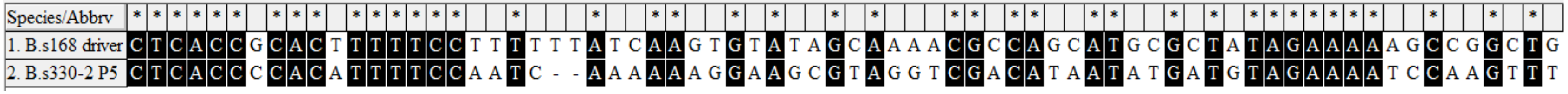


33. Putative capsular polysaccharide biosynthesis protein (*ywqC*)

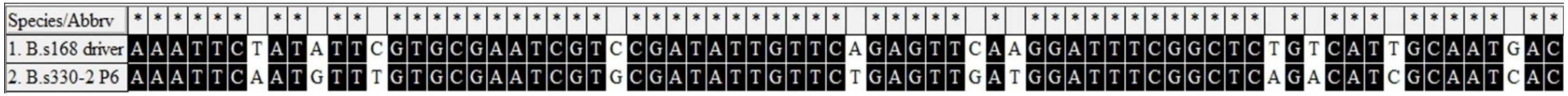


34. N-acetylmuramoyl-L-alanine amidase (*cwlB*)

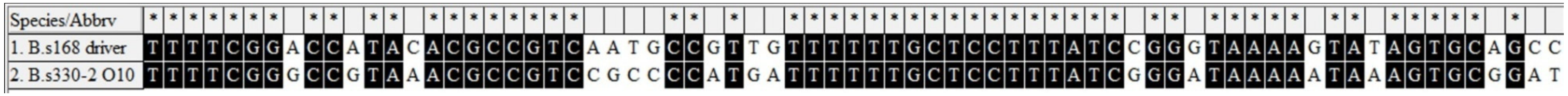


35. Phosphofructokinase

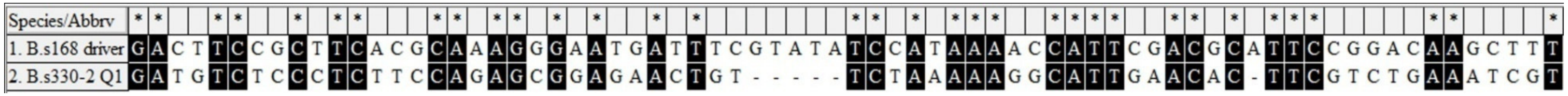

36. Beta-lactamase (*penP*)

| Species/Abbrv | ******* *********** ****** *******  ***** ************************ |
|---|---|
| 1. B.s168 driver | TGGCAATTCTGTCAAACCGCTTTACGAAAGATGCTAATTATGATAATGCTCTTATCGCTGAGGCTGCAAAAG |
| 2. B.S330-2 Q3 | TGGCAATCCTGTCAAACCGATTTACGGAAGATGCCGATTATCATAATGCTCTTATCGCTGAGGCTG------ |

37. S-adenosylmethionine synthetase (*metK*)

| Species/Abbrv | *     ***        ***       ***  **  ** ** *** **** *  *****     ** * *** |
|---|---|
| 1. B.s168 driver | TGGGATAACAGGGACAAACGGCAAAACATCAACGACCCACATGGTCGATGAGATTCTGAAAAAAGCGGGAAA |
| 2. B.s330-2 Q6 | TTCATCAACCCTACGGGACGTTTCGTAATCGGCGGGCCTCAAGGT-GATGCGGGTCTGACC--GGCCGTAAA |

38. Macrolactin (*mlnF*)

| Species/Abbrv | *******   ** * * **** * ***** *         ** **  ** *****   **  * ** *** * |
|---|---|
| 1. B.s168 driver | GATGGCGATGAGAAGGATTCACACTGATATGCCGGATCTCAACCTCAGAATCCTTTTTTTCGACTCCGATTG |
| 2. B.s330-2 Q10 | GATGGCG-CAAGGATGGTTCA-ATTGATAGG---------AATCTTTGAGTCCTTGAATTTCAATCGGATCG |

39. Aspartokinase II (*lysC*)

| Species/Abbrv | ******  *  **   ****     *  * ****     * *  ***** ** *   * **    ******* |
|---|---|
| 1. B.s168 driver | GACGCCTGCATTTCAGCGGG---AAATACCGGTGCATTAATGACAGCCGGTCTCTTTATTGTCGGGAGAATT |
| 2. B.s330-2 R1 | GACGCCGTCTCTTATACGGGCTGGCAGGCGGGTGTCCGCACGGAAGCCGTTCACGGCAATGC---GAGAATT |

40. Formate dehydrogenase (*yjgC*)

| Species/Abbrv | ************  * * ** **     *   ***   **    ** *  * * ***     *   * * ** |
|---|---|
| 1. B.s168 driver | GCGGCGTCTTTTACACCCTTTATACACCGGATGACCGATTCAATTTCTTTCGTTTCTACACGGTATCCTCTG |
| 2. B.s330-2 R3 | GCGGCGTCTTTTGAATCGTTCATCGGGAGATAGACTTGTTTGCCTTTTACCCTGTCTGTGATGACGCATTTG |

41. Transport protein (*tqsA*)

| Species/Abbrv | ***** ****  * *   *** ** * * *  ***     *    **  *   * *** * ** *    ** |
|---|---|
| 1. B.s168 driver | CGTTTGCTTGATGCCGGAATTAAAGGAAAGGAATCCATTCCGCAGGTGACAAAGAGCTCGGTTGTCGGCATT |
| 2. B.s330-2 R7 | CGTTT-CTTGCCGGCACGATTGAAAGCACG--ATCAGAAGCATCCGTTTC---GTGCTTGATTAT----ATC |

42. Dihydroorotase (*pyrC*)

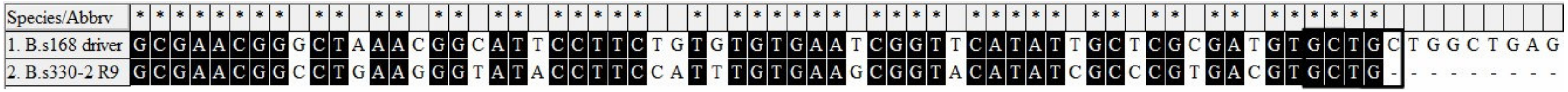


43. Alkaline phosphatase synthesis sensor protein (*phoR*)

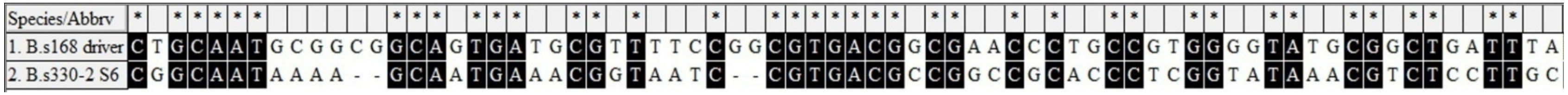


44. Cell wall-associated protein (*wapA*)

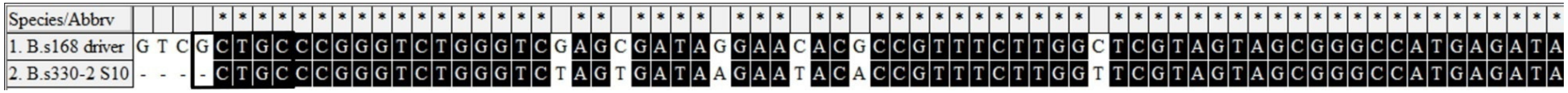


45. Iturin A (*ituA*)

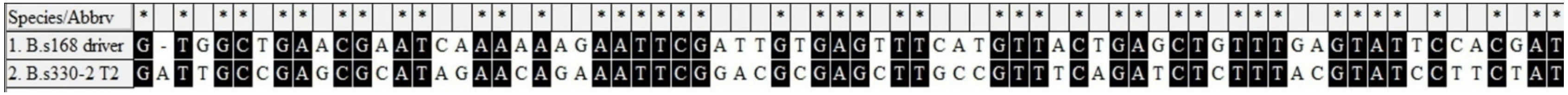


46. Flagellin protein (*hag*)

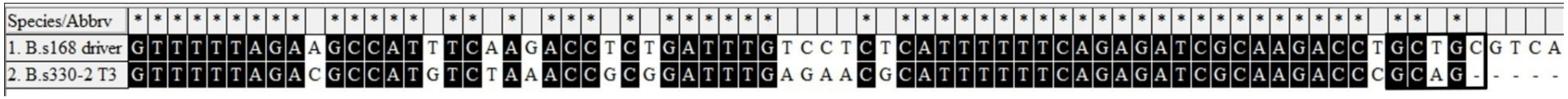


47. NADH-dependent butanol dehydrogenase A (*yugJ*)

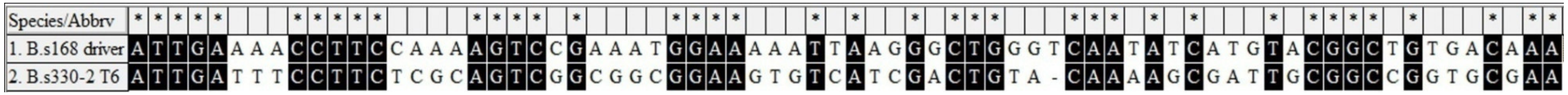

48. Cystathionine beta-lyase (*metC*)

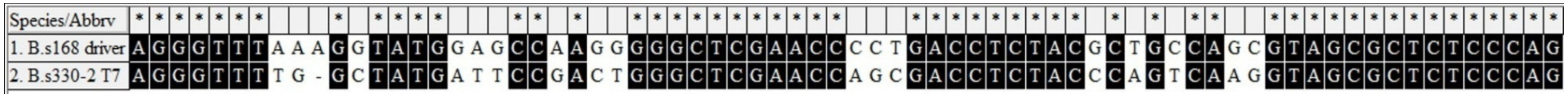


49. Cell-division protein (*ftsH*)

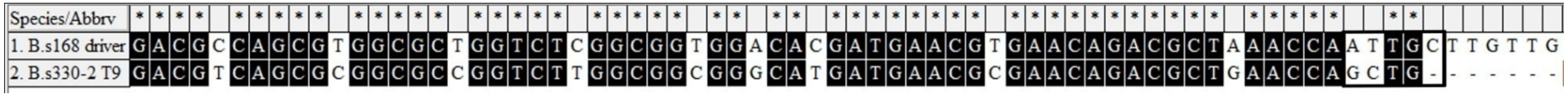


50. Gamma-glutamyltransferase (*ggt*)

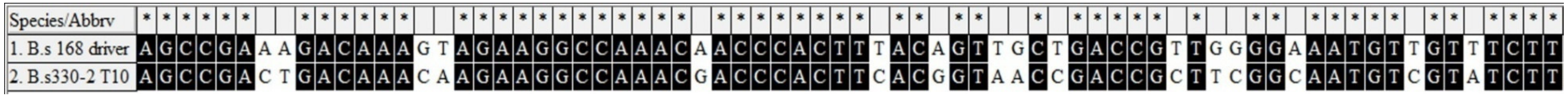


51. Anthranilate synthase component 2 (*pabA*)

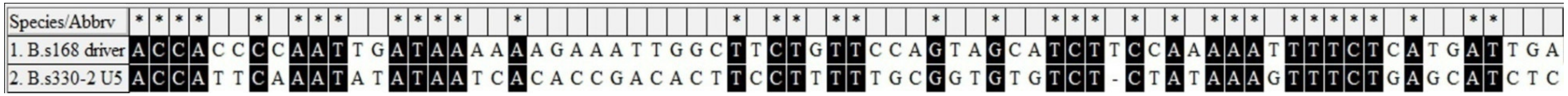


52. Putative acyltransferase (*yfiQ*)

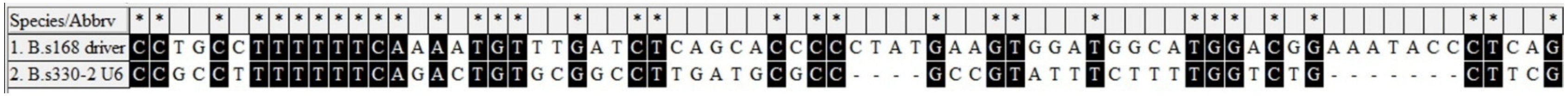


53. Glycolate oxidase (*glcD* )

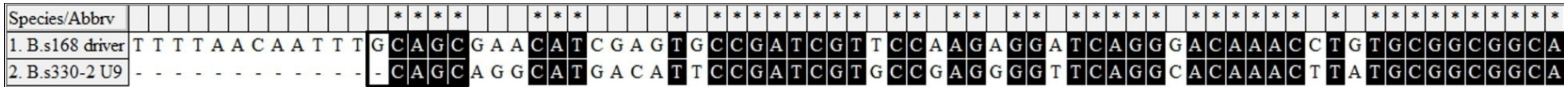

54. Nitrate reductase subunit gamm (*narI*)

| Species/Abbrv | ***************** ***************************** ******************** |
| --- | --- |
| 1. B.s168 driver | TAATCAAATCCTTTCGAATCAATGTTGAGAAACGTGGCCGCAACGCCTGACAGCATCATGAACAGCAGCAGG |
| 2. B.s330-2 W3 | TAATCAAATCCTTTCGAGTCAATGTTGAGAAACGTGGCCGCAACGCCCGACAGCATCATGAACAGCAG---- |

55. Penicillin-binding protein 2B (*pbpB*)

| Species/Abbrv | ** *  ********  * ****** * ** **  * *  *** * *** **** *** * **  ******** |
| --- | --- |
| 1. B.s168 driver | CAGACAATGCCTGATTTAACAGATCAAACGGTAGCTGCTGCTCAAAAGAAAGCAAAAGAGGAGAATCTCACA |
| 2. B.s330-2 W9 | CAAAAGATGCCTGACCTGACAGATAAGACAGTCACGGGCGCTGAGAAGGAAGCGAAAAATGAACATCTCACA |

56. Elongation factor Tu (*tuf*)

| Species/Abbrv | ************* *********************************************** |
| --- | --- |
| 1. B.s168 driver | AGTACGAAACTGAAACTCGTCACTATGCACACGTTGACTGCCCAGGACACGCTGACTATGTTAAAAACATGA |
| 2. B.s330-2 X2 | AGTACGAAACTGACACTCGTCACTATGCACACGTTGACTGCCCAGGACACGCTGACTATGT----------- |
